# Supplementary material for: Real-space machine learning of correlation density functionals
Source: Nat Commun. 2025 Dec 1;16:11306. doi: 10.1038/s41467-025-66450-z (PMC12722768; doi:10.1038/s41467-025-66450-z)
Supplement: Supplementary file 1 — Supplementary Infomation [file 41467_2025_66450_MOESM1_ESM.pdf]

Supplementary Information for: Real-space machine learning of correlation density functionals

Elias Polak[1], Heng Zhao[1], and Stefan Vuckovic[1,*)]

[1] *Department of Chemistry, University of Fribourg, CH-1700 Fribourg, Switzerland.*

[*)] Corresponding author: stefan.vuckovic@unifr.ch

CONTENTS

S1. MP2-based correlation energy density 3

S2. Effect of regularization on MP2 results 4

S3. Implementation techniques of the MP2 correlation energy density generator 5

S4. Additional $\Delta P_2^{\mathbf{MP2}}(\mathbf{r}, \mathbf{r}')$ plots for stretched helium dimer 7

S5. Regularized MP2 interaction energies 8

S6. ML features for ML2 and MLS2 9

S7. Scaling invariance of the MP2 correlation energy density 11

S8. Loss functions for ML2 and MLS2 13

S9. Additional results for ML2 14

S10. Training set size dependence on ML2 transferability to diatomics 22

S11. Scaling of MLS2 weights 24

S12. Training and test datasets of MLS2 25

S13. Training and test datasets of MLS2@W4 27

S14. Additional Plots for ML(S)2 training details 30

Supplementary References 30

S1. MP2-BASED CORRELATION ENERGY DENSITY

In this section, we give a brief summary of how how our recently introduced Møller–Plesset adiabatic connection (MPAC) correlation energy density framework[1] yields the MP2 correlation energy density per particle (Eq. 5). Consider the following coupling constant λ-dependent Hamiltonian[1,2] for N-electron system:

$$\hat{H}_\lambda = \hat{T} + \hat{V}_{\text{ext}} + \lambda \hat{V}_{ee} + (1-\lambda)(\hat{J} + \hat{K}), \tag{S1}$$

where $\hat{T}$ is the kinetic energy operator, $\hat{V}_{\text{ext}}$ the external potential, $\hat{V}_{ee}$ the electron-electron interaction, and $\hat{J}$ and $\hat{K}$ are the standard HF Coulomb and exchange operators, defined in terms of the HF density and orbitals, respectively. Let Ψ_λ be the ground-state wavefunction of $\hat{H}_\lambda$. Then, the MPAC integral expression for correlation energy (true - HF energy) reads,

$$E_{\text{c}} = \int_0^1 E_{\text{c}}^\lambda d\lambda, \tag{S2}$$

where E_{c}^λ is defined as

$$E_{\text{c}}^\lambda = \left\langle \Psi_\lambda \middle| \hat{V}_{ee} - \hat{J} - \hat{K} \middle| \Psi_\lambda \right\rangle - \left\langle \Psi_0 \middle| \hat{V}_{ee} - \hat{J} - \hat{K} \middle| \Psi_0 \right\rangle. \tag{S3}$$

In Ref. 1, the expression for $e_{\text{c}}^\lambda(\mathbf{r})$ was derived using a gauge analogous to the correlation hole in DFT, which, upon λ-integration [Eq. S2], yields the corresponding $e_{\text{c}}(\mathbf{r})$,

$$E_{\text{c}} = \int e_{\text{c}}(\mathbf{r})\rho(\mathbf{r})d\mathbf{r} = \int \left[\int_0^1 e_{\text{c}}^\lambda(\mathbf{r})d\lambda\right]\rho(\mathbf{r})d\mathbf{r}. \tag{S4}$$

Expanding $e_{\text{c}}^\lambda(\mathbf{r})$ in the small-λ limit (see Ref. 1 for further details), yields,

$$e_{\text{c}}^\lambda(\mathbf{r}) \approx e_{\text{c}}'(\mathbf{r})\lambda = \frac{1}{2\rho(\mathbf{r})}\int \frac{P_2'(\mathbf{r},\mathbf{r}')}{|\mathbf{r}-\mathbf{r}'|}d\mathbf{r}'\lambda \qquad \text{for } \lambda \to 0, \tag{S5}$$

where,

$$e_{\text{c}}'(\mathbf{r}) = \frac{d}{d\lambda} e_{\text{c}}^\lambda(\mathbf{r})\bigg|_{\lambda=0} \tag{S6a}$$

$$P_2^{\text{MP2}}(\mathbf{r},\mathbf{r}') = P_2'(\mathbf{r},\mathbf{r}') = \frac{d}{d\lambda} P_2^\lambda(\mathbf{r},\mathbf{r}')\bigg|_{\lambda=0} \tag{S6b}$$

$$P_2^\lambda(\mathbf{r},\mathbf{r}') = N(N-1)\int |\Psi_\lambda(\mathbf{r},\mathbf{r}',\mathbf{r}_3,\ldots,\mathbf{r}_N)|^2\, d\mathbf{r}_3\cdots d\mathbf{r}_N \tag{S6c}$$

with $P_2^\lambda(\mathbf{r},\mathbf{r}')$ being the λ- dependent pair density, where real Ψ_λ is assumed in Eq. S6c. By virtue of Eq. S6b, $P_2^{\text{MP2}}(\mathbf{r},\mathbf{r}') = P_2'(\mathbf{r},\mathbf{r}')$ corresponds to the first-order derivative of the pair density $P_2^\lambda(\mathbf{r},\mathbf{r}')$ with respect to λ, evaluated at $\lambda = 0$. Therefore, it contains only the correlation component, and resolving it in terms of HF orbitals yields[1],

$$P_2^{\text{MP2}}(\mathbf{r},\mathbf{r}') = -2\sum_{ijab} t_{ij}^{ab}\big(\phi_i(\mathbf{r})\phi_j(\mathbf{r}')\phi_a(\mathbf{r}')\phi_b(\mathbf{r})\delta_{ia}\delta_{jb} - \phi_i(\mathbf{r})\phi_j(\mathbf{r})\phi_a(\mathbf{r}')\phi_b(\mathbf{r}')\delta_{ib}\delta_{ja}\big), \tag{S7}$$

where δ is the Kronecker δ over two spin indices and t_{ij}^{ab} are the MP2 double amplitudes, $t_{ij}^{ab} = T_{ijab}\delta_{ia}\delta_{jb} - T_{ijba}\delta_{ib}\delta_{ja}$, with $T_{ijab} = \frac{\langle ij|ab\rangle}{\varepsilon_a+\varepsilon_b-\varepsilon_i-\varepsilon_j}$ defined as the partial MP2 double amplitudes in Eq. (7) using orbital energies, ε, and two-electron integrals, $\langle ij|ab\rangle$. Finally, taking the λ-integral from Eq. S4 of Eq. S5 yields,

$$e_{\mathrm{c}}(\mathbf{r}) \approx \frac{1}{2}e'_{\mathrm{c}}(\mathbf{r}) = e_{\mathrm{c}}^{\mathrm{MP2}}(\mathbf{r}) = \frac{1}{4\rho(\mathbf{r})}\int \frac{P_2^{\mathrm{MP2}}(\mathbf{r},\mathbf{r}')}{|\mathbf{r}-\mathbf{r}'|}d\mathbf{r}'. \tag{S8}$$

Applying the κ-regularization[3,4] to the partial MP2 double amplitudes (see Eq. (9)),

$$T_{ijab}^{\kappa} = T_{ijab}\left(1 - e^{-\kappa(\varepsilon_a+\varepsilon_b-\varepsilon_i-\varepsilon_j)}\right)^2,$$

results in $P_2^{\kappa\mathrm{MP2}}(\mathbf{r},\mathbf{r}')$, which is the regularized expression of Eq. S7, and thus, we have $e_{\mathrm{c}}^{\kappa\mathrm{MP2}}(\mathbf{r})$ from Eq. 10.

S2. EFFECT OF REGULARIZATION ON MP2 RESULTS

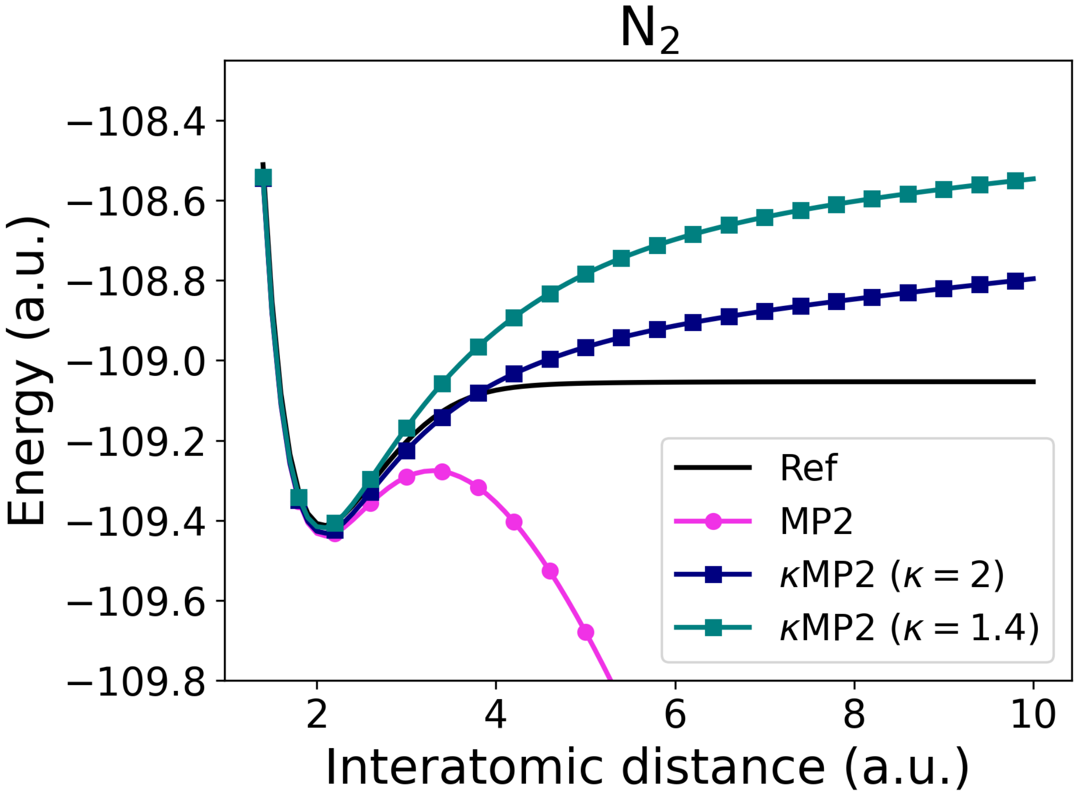

Fig. S1: Dissociation energy curve of N_2 at different κ values for κ-regularized second-order Møller–Plesset perturbation theory (κMP2). MP2 corresponds to $\kappa \to \infty$. Reference (Ref) taken from Ref. 5.

S3. IMPLEMENTATION TECHNIQUES OF THE MP2 CORRELATION ENERGY DENSITY GENERATOR

This section is devoted to the numerical settings of our MP2 correlation energy density implementation in the Python coding language. The resulting generator reads the relevant atomic orbital functions from a HF treatment using the PySCF library[6,7] and performs the necessary MP2-based integrations and tensor contractions. This procedure scales similar to other second-order perturbation theory derived models, and thus, we can employ some approximation techniques to enhance the applicability and efficiency of our code. The resulting accuracy can be assessed using the correlation energy result from the already implemented MP2 module in PySCF (refMP2).

For the following discussion, we introduce some notation for the orbitals:

- the molecular orbital functions (MOs) ϕ_i;
- the atomic orbital functions (AOs) χ_n;
- the auxiliary basis functions ψ_t.

Expanding the MOs inside the integral of $V_{ijab}(\mathbf{r})$ in AO basis, $\phi_i(\mathbf{r}) = \sum_n C_{ni}\chi_n(\mathbf{r})$, yields

$$\begin{aligned} V_{ijab}(\mathbf{r}) &= \phi_i(\mathbf{r})\phi_a(\mathbf{r}) \sum_{m,n} C_{mj}C_{nb} \int \frac{\chi_m(\mathbf{r}')\chi_n(\mathbf{r}')}{|\mathbf{r}-\mathbf{r}'|} d\mathbf{r}' \\ &= \phi_i(\mathbf{r})\phi_a(\mathbf{r}) \sum_{m,n} C_{mj}C_{nb}A_{mn}(\mathbf{r}), \end{aligned} \tag{S9}$$

where we define the tensor integral

$$A_{mn}(\mathbf{r}) = \int \frac{\chi_m(\mathbf{r}')\chi_n(\mathbf{r}')}{|\mathbf{r}-\mathbf{r}'|} d\mathbf{r}'.$$

Density Fitting[8,9] (DF) is a common strategy for MP2 to improve the scaling of the two-electron integral evaluations. It uses an auxiliary basis to expand the direct product of AOs, $\chi_m(\mathbf{r})\chi_n(\mathbf{r}) = \sum_t Q_{mnt}\psi_t(\mathbf{r})$. This allows a one order of magnitude speed increase of our code since the resulting integral evaluation only scales linearly with the basis set (or number of electrons). Specifically, the orbital potential $V_{ijab}(\mathbf{r})$ from Eq. S9 then becomes

$$V_{ijab}(\mathbf{r}) = \phi_i(\mathbf{r})\phi_a(\mathbf{r}) \sum_{m,n,t} C_{mj}C_{nb}Q_{mnt} \int \frac{\psi_t(\mathbf{r}')}{|\mathbf{r}-\mathbf{r}'|} d\mathbf{r}'. \tag{S10}$$

Employing a tensor notation for the integral

$$I_t(\mathbf{r}) = \int \frac{\psi_t(\mathbf{r}')}{|\mathbf{r}-\mathbf{r}'|} d\mathbf{r}'$$

results in the following density fitted orbital potential formulation:

$$V_{ijab}(\mathbf{r}) = \phi_i(\mathbf{r})\phi_a(\mathbf{r}) \sum_{m,n,t} C_{mj}C_{nb}Q_{mnt}I_t(\mathbf{r}). \tag{S11}$$

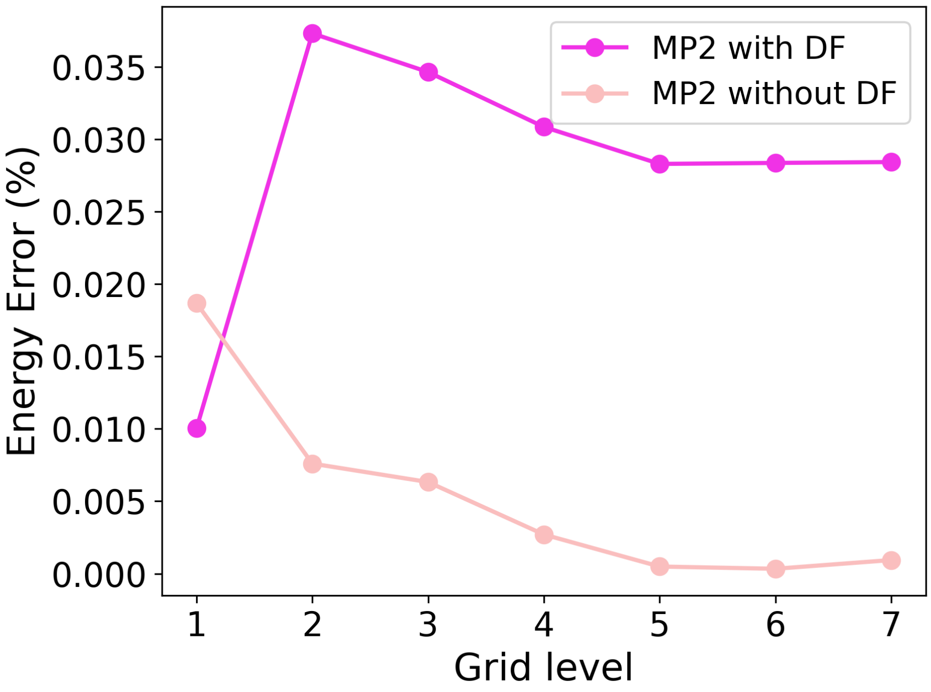

Fig. S2: Relative interaction energy error. The shown data is evaluated with respect to the PySCF-based second-order Møller–Plesset perturbation theory (refMP2) value (-0.0029 kcal mol^{-1}) at different density functional theory discretization grid-levels[10] for Ne_2 at equilibrium (geometry taken from Ref. 11). Plotted are the results with and without the density fitting (DF) approximation.

We assess correlation energy error due to DF using interaction energies. For example, the relative absolute interaction energy error with respect to refMP2 for Ne_2 is plotted in Fig. **S2** at different DFT discretization grid levels[10].

As expected, the error in correlation energy with respect to refMP2 vanishes as the grid size increases. It shows furthermore how the default grid-level (3) is already very accurate with an error below 0.01% in the interaction energy. Using the DF approximation results in a constant shift with higher integration accuracy. Nevertheless, this difference is still negligible relative to the refMP2 interaction energy. Throughout the rest of this work, we always employ the DF approximation, expecting negligible errors arising from it.

S4. ADDITIONAL $\Delta P_2^{\mathbf{MP2}}(\mathbf{r}, \mathbf{r}')$ PLOTS FOR STRETCHED HELIUM DIMER

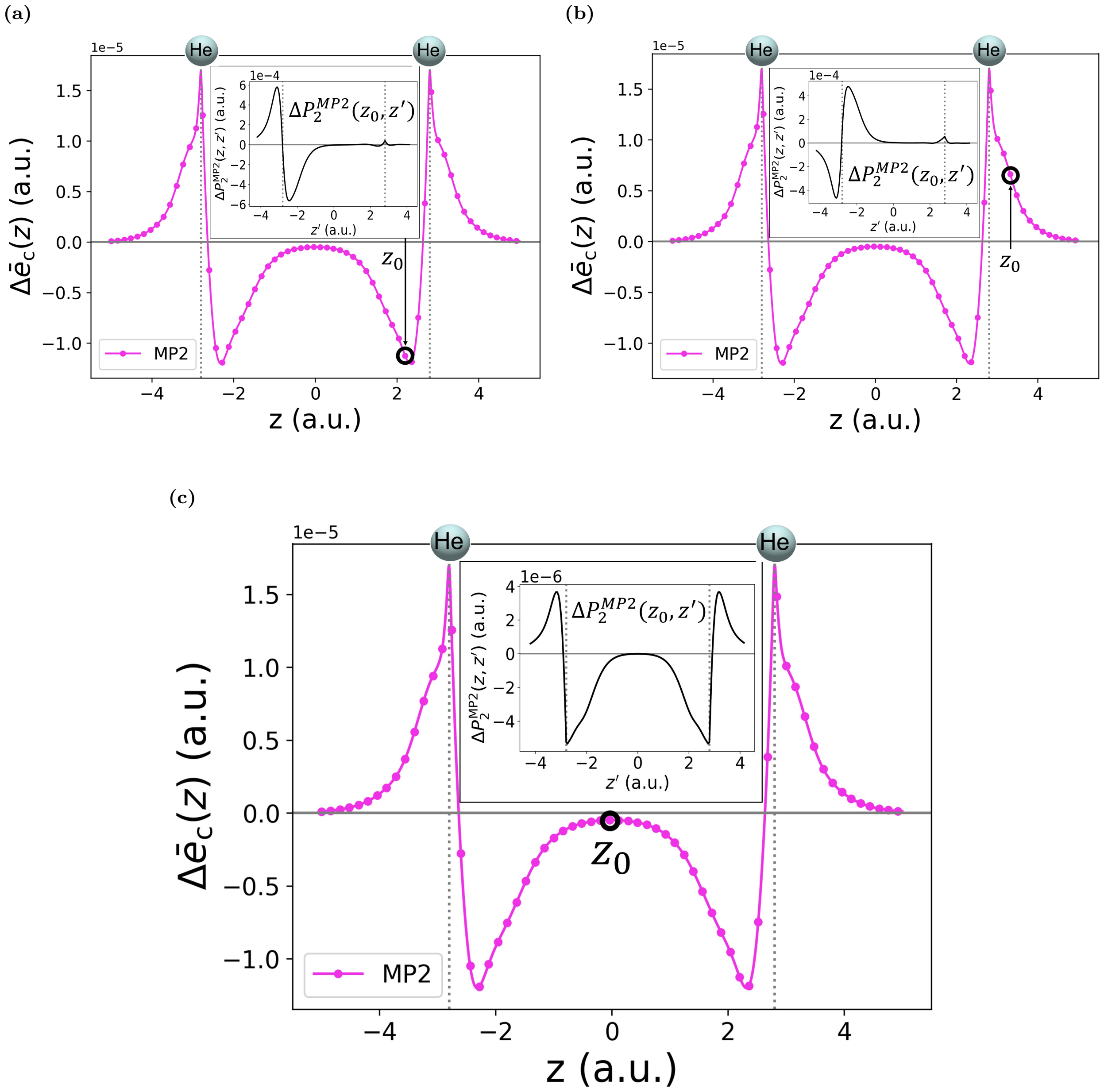

Fig. S3: Visualization of $\Delta\bar{e}_c^{\mathrm{MP2}}(z)$ (total correlation energy density minus the one from the individual subsystem) for second-order Møller–Plesset perturbation theory (MP2) as in Fig. **2a**. Panels **S3a–S3c** differ in their interaction pair density $\Delta P_2^{\mathrm{MP2}}(z_0, z')$ inset plots, which are given at three different z_0 positions.

S5. REGULARIZED MP2 INTERACTION ENERGIES

κ	0.2	0.4	0.6	0.8	1.0	1.2	1.4	2.0	∞
$E^{\kappa\text{MP2}}$	0.0089	0.0011	-0.0024	-0.0044	-0.0057	-0.0065	-0.0070	-0.0073	-0.0074

Table S1: Interaction energies (kcal mol^{-1}) of the helium dimer for different κ-regularizations of second-order Møller–Plesset perturbation theory (κMP2). Data corresponds to the energy density plots in Figs. **1(d,top)** and **2a**. $\kappa \to \infty$ represents MP2 without regularization. Additional interaction energies for comparison: Coupled-Cluster with singles, doubles, and perturbative triples (CCSD(T)), $E^{\text{CCSD(T)}} = -0.013$ and Hartree-Fock (HF), $E^{\text{HF}} = 0.018$.

Method	Ref	HF	MP2	κMP2
E	-1.5	1.13	-1.87	-1.58

Table S2: Interaction energies (kcal mol^{-1}) of the benzene-methane complex. Data corresponds to the interaction energy density visualizations in Figs. **2b**– **2d**. Reference value (Ref) taken from the S22 database[12]. Energies from Hartree-Fock (HF), second-order Møller–Plesset perturbation theory (MP2) and its κ-regularized counterpart (κMP2) are shown for comparison.

S6. ML FEATURES FOR ML2 AND MLS2

We list in this section formulas of the DFT-based ML features that were used for the training of the ML2 and the MLS2 models. They are all implemented in Python as dimensionless objects and then evaluated at every point of the DFT grid for the input data.

ML features for ML2:

- Reduced density gradient,

$$s(\mathbf{r}) = \frac{|\nabla\rho(\mathbf{r})|}{2\,(3\pi^2)^{1/3}\,\rho^{4/3}(\mathbf{r})}.$$

- Reduced density Laplacian,

$$q(\mathbf{r}) = \frac{\nabla^2\rho(\mathbf{r})}{4\,(3\pi^2)^{2/3}\,\rho^{5/3}(\mathbf{r})}.$$

- Regularized kinetic energy variable from the r^2SCAN DFA[13],

$$\alpha(\mathbf{r}) = \frac{\tau(\mathbf{r}) - \tau_{\mathrm{w}}(\mathbf{r})}{3\,(3\pi^2)^{2/3}\,\rho^{5/3}(\mathbf{r})/10 + \eta\tau_{\mathrm{w}}(\mathbf{r})},$$

  where $\eta = 10^{-3}$, and

$$\tau(\mathbf{r}) = \frac{1}{2}\sum_i |\nabla\phi_i(\mathbf{r})|^2 \text{ and } \tau_{\mathrm{w}}(\mathbf{r}) = \frac{|\nabla\rho(\mathbf{r})|^2}{8\rho(\mathbf{r})}$$

  are the kinetic energy density and the von Weizsäcker kinetic energy density.

- Fractional occupation number weighted density[14,15] (FOD) normalized by the density,

$$\rho^{\mathrm{FOD}}(\mathbf{r}) = \frac{1}{\rho(\mathbf{r})}\sum_i(\delta_1 - \delta_2 f_i)|\phi_i(\mathbf{r})|^2,$$

  where δ_1 and δ_2 are chosen such that only fractionally occopied ϕ_i contribute to the sum. The weights f_i are given by the Fermi-Dirac distribution,

$$f_i = \frac{1}{e^{(\varepsilon_i - E_F)/kT} + 1}.$$

  Here, ε_i are orbital energies, E_F is the Fermi energy and $k = 3.166811563 \times 10^{-6}$ is the Boltzmann constant in Hartree per Kelvin. The electronic temperature T is set to $T_1 = 10000K$ or $T_2 = 25000K$, yielding two different FOD features for the neural network, ρ_1^{FOD} and ρ_2^{FOD}.

Additional features for MLS2 (on top of the ones used for ML2):

- Wigner-Seitz radius,

$$r_s(\mathbf{r}) = \left(\frac{3}{4\pi\rho(\mathbf{r})}\right)^{1/3}.$$

- Normalized κMP2 correlation energy density,

$$\tilde{e}_{\mathrm{c}}^{\kappa\mathrm{MP2}}(\mathbf{r}) = \frac{e_{\mathrm{c}}^{\kappa\mathrm{MP2}}(\mathbf{r})}{\rho^{-1/3}(\mathbf{r})e_{\mathrm{x}}(\mathbf{r})},$$

  where $e_{\mathrm{x}}(\mathbf{r})$ is the exchange energy density coming from the same gauge as $e_{\mathrm{c}}^{\mathrm{MP2}}(\mathbf{r})$.

- Normalized os-κMP2 correlation energy density,

$$\tilde{e}_{\mathrm{c,os}}^{\kappa\mathrm{MP2}}(\mathbf{r}) = \frac{e_{\mathrm{c,os}}^{\kappa\mathrm{MP2}}(\mathbf{r})}{\rho^{-1/3}(\mathbf{r})e_{\mathrm{x}}(\mathbf{r})}.$$

- Spin polarization (for open-shell systems),

$$\zeta(\mathbf{r}) = \frac{\rho_\alpha(\mathbf{r}) - \rho_\beta(\mathbf{r})}{\rho(\mathbf{r})}, \tag{S12}$$

  defined in terms of the the spin densities $\rho_\alpha(\mathbf{r})$ and $\rho_\beta(\mathbf{r})$.

S7. SCALING INVARIANCE OF THE MP2 CORRELATION ENERGY DENSITY

In this section, we derive the scaling invariance of the MP2 correlation energy density defined in Eq. 6 once evaluated on either KS or HF orbitals.

Uniform density scaling[16] scales the density with respect to a scalar $\gamma > 0$ as follows: $\rho_\gamma(\mathbf{r}) = \gamma^3\rho(\gamma\mathbf{r})$. A direct consequence is the scaling of the corresponding orbitals:

$$\phi[\rho_\gamma](\mathbf{r}) = \gamma^{3/2}\phi[\rho](\gamma\mathbf{r}). \tag{S13}$$

For the scaling invariance of $e_{\mathrm{c}}^{\mathrm{MP2}}(\mathbf{r})$, we need to show that the only real-valued exponent p for which

$$e_{\mathrm{c}}^{\mathrm{MP2}}[\rho_\gamma](\mathbf{r}) = \gamma^p e_{\mathrm{c}}^{\mathrm{MP2}}[\rho](\gamma\mathbf{r})$$

holds, is $p = 0$ (see Eq. 13).

First, we derive the scaling of T_{ijab} from Eq. 7. Its nominator has the same linear scaling ($p = 1$) as the electron-electron interaction density functional[16], while the denominator depends on the scaling of orbital energies (see Ref. 17):

$$\varepsilon_i[\rho_\gamma] = \gamma^2\varepsilon_i[\rho] \tag{S14}$$

Combining now the scaling of the nominator and of the denominator of Eq. 7 gives

$$\begin{aligned} T_{ijab}[\rho_\gamma] &= \frac{\gamma\langle ij|ab\rangle}{\varepsilon_i[\rho_\gamma] + \varepsilon_j[\rho_\gamma] - \varepsilon_a[\rho_\gamma] - \varepsilon_b[\rho_\gamma]} \\ &= \frac{\gamma\langle ij|ab\rangle}{\gamma^2\varepsilon_i[\rho] + \gamma^2\varepsilon_j[\rho] - \gamma^2\varepsilon_a[\rho] - \gamma^2\varepsilon_b[\rho]} \\ &= \gamma^{-1}\frac{\langle ij|ab\rangle}{\varepsilon_i[\rho] + \varepsilon_j[\rho] - \varepsilon_a[\rho] - \varepsilon_b[\rho]} \\ &= \gamma^{-1}T_{ijab}[\rho], \end{aligned} \tag{S15}$$

which shows the $p = -1$ scaling property of T_{ijab}.

Next, we derive the scaling of $V_{ijab}(\mathbf{r})$ from Eq. 8 using again the scaling property of orbitals and the transformation of the integral variable:

$$\begin{aligned} V_{ijab}[\rho_\gamma](\mathbf{r}) &= \phi_i[\rho_\gamma](\mathbf{r})\phi_j[\rho_\gamma](\mathbf{r})\int\frac{\phi_a[\rho_\gamma](\mathbf{r}')\phi_b[\rho_\gamma](\mathbf{r}')}{|\mathbf{r}-\mathbf{r}'|}d\mathbf{r}' \\ &= \gamma^{3/2}\phi_i[\rho](\gamma\mathbf{r})\gamma^{3/2}\phi_j[\rho](\gamma\mathbf{r})\int\frac{\gamma^{3/2}\phi_a[\rho](\gamma\mathbf{r}')\gamma^{3/2}\phi_b[\rho](\gamma\mathbf{r}')}{|\mathbf{r}-\mathbf{r}'|}d\mathbf{r}' \\ &= \gamma^3\phi_i[\rho](\gamma\mathbf{r})\phi_j[\rho](\gamma\mathbf{r})\int\frac{\phi_a[\rho](\gamma\mathbf{r}')\phi_b[\rho](\gamma\mathbf{r}')}{|\gamma\mathbf{r}-\gamma\mathbf{r}'|}\gamma^4 d\mathbf{r}' \\ &= \gamma^3\phi_i[\rho](\gamma\mathbf{r})\phi_j[\rho](\gamma\mathbf{r})\gamma\int\frac{\phi_a[\rho](\mathbf{s})\phi_b[\rho](\mathbf{s})}{|\gamma\mathbf{r}-\mathbf{s}|}d\mathbf{s} \\ &= \gamma^4 V_{ijab}[\rho](\gamma\mathbf{r}). \end{aligned} \tag{S16}$$

Finally, combining the results for T_{ijab} ($p = -1$) from Eq. S15 and for $V_{ijab}(\mathbf{r})$ ($p = 4$) from Eq. S16 with the uniform density scaling ($p = 3$), concludes the scaling invariance of the

MP2 correlation energy density:

$$
\begin{aligned}
e_{\mathrm{c}}^{\mathrm{MP2}}[\rho_\gamma](\mathbf{r}) &= -\frac{1}{4\rho_\gamma(\mathbf{r})}\sum_{ijab}\left[(T_{ijab}[\rho_\gamma]\delta_{ia}\delta_{jb} - T_{ijba}[\rho_\gamma]\delta_{ib}\delta_{ja})(V_{ijab}[\rho_\gamma](\mathbf{r})\delta_{ia}\delta_{jb} - V_{ijba}[\rho_\gamma](\mathbf{r})\delta_{ib}\delta_{ja}\right] \\
&= -\frac{1}{4\gamma^3\rho(\gamma\mathbf{r})}\sum_{ijab}\left[(\gamma^{-1}T_{ijab}[\rho]\delta_{ia}\delta_{jb} - \gamma^{-1}T_{ijba}[\rho]\delta_{ib}\delta_{ja})(\gamma^4 V_{ijab}[\rho](\gamma\mathbf{r})\delta_{ia}\delta_{jb}\right. \\
&\quad \left. -\gamma^4 V_{ijba}[\rho](\gamma\mathbf{r})\delta_{ib}\delta_{ja}\right] \\
&= -\frac{1}{4\rho(\gamma\mathbf{r})}\sum_{ijab}\left[(T_{ijab}[\rho]\delta_{ia}\delta_{jb} - T_{ijba}[\rho]\delta_{ib}\delta_{ja})(V_{ijab}[\rho](\gamma\mathbf{r})\delta_{ia}\delta_{jb} - V_{ijba}[\rho](\gamma\mathbf{r})\delta_{ib}\delta_{ja}\right] \\
&= e_{\mathrm{c}}^{\mathrm{MP2}}[\rho](\gamma\mathbf{r}).
\end{aligned} \tag{S17}
$$

The same scaling invariance also applies to $e_{\mathrm{c}}^{\kappa\mathrm{MP2}}$ and to its spin-resolved components.

S8. LOSS FUNCTIONS FOR ML2 AND MLS2

For the LES-based training of the ML2 neural network, we employ the mean of $\mathcal{L}_{\text{LES}}$ from Eq. 3 scaled by the number of electrons:

$$\text{Mean(LES)} = \frac{1}{M}\sum_{k=1}^{M}\frac{\mathcal{L}_{\text{LES}}^{k}}{N_k}, \tag{S18}$$

where M is the number of systems ($M = 8$ in our case: H^-, He, Be, Mg, Ne, Ar, Ca, and Kr) and N_k indicates the number of electrons of the k-th system with $\mathcal{L}_{\text{LES}}^{k}$ being the corresponding k-th LES.

The GES-based training of the ML2 neural network uses a similar mean of $\mathcal{L}_{\text{GES}}$ from Eq. 2:

$$\text{Mean(GES)} = \frac{1}{M}\sum_{k=1}^{M}\mathcal{L}_{\text{GES}}^{k}, \tag{S19}$$

where $\mathcal{L}_{\text{GES}}^{k}$ is the GES of the k-th system.

In the case of MLS2, we employ a GES combined with the interaction correlation energy error. First, the mean absolute relative error (MArE) is calculated via

$$\text{MArE} = \frac{1}{M}\sum_{k=1}^{M}\frac{\mathcal{L}_{\text{GES}}^{k}}{|E_{\text{c}}^{\text{ref}}[\rho_k]|}, \tag{S20}$$

with the density ρ_k indicating the reference correlation energy value of the k-th system. Second, we evaluate interaction GES ($\mathcal{L}_{\text{int-GES}}$) using reference interaction energies ($E_{\text{c}}^{\text{int-Ref}}$) and employ the corresponding mean absolute relative interaction error (int-MArE) as

$$\text{int-MArE} = \frac{1}{M}\sum_{k=1}^{M}\frac{\mathcal{L}_{\text{int-GES}}^{k}}{|E_{\text{c}}^{\text{int-ref}}[\rho_k]|}. \tag{S21}$$

The final total loss function is the average between MArE from Eq. S20 and int-MArE from Eq. S21.

S9. ADDITIONAL RESULTS FOR ML2

In this section, additional data on validation, convergence, uniqueness and robustness are presented for the GES-based and LES-based ML2 models.

As noted in the main paper, we use eight small closed-shell atoms/ions (H^-, He, Be, Mg, Ne, Ar, Ca, and Kr) to train ML2. For ML2's validation, we select ions of similar size to the training set (Li^+, Ca^{2+}, and Ne^{2+}). Fig. **S4** shows that the validation loss closely follows the training loss for LES-based ML2.

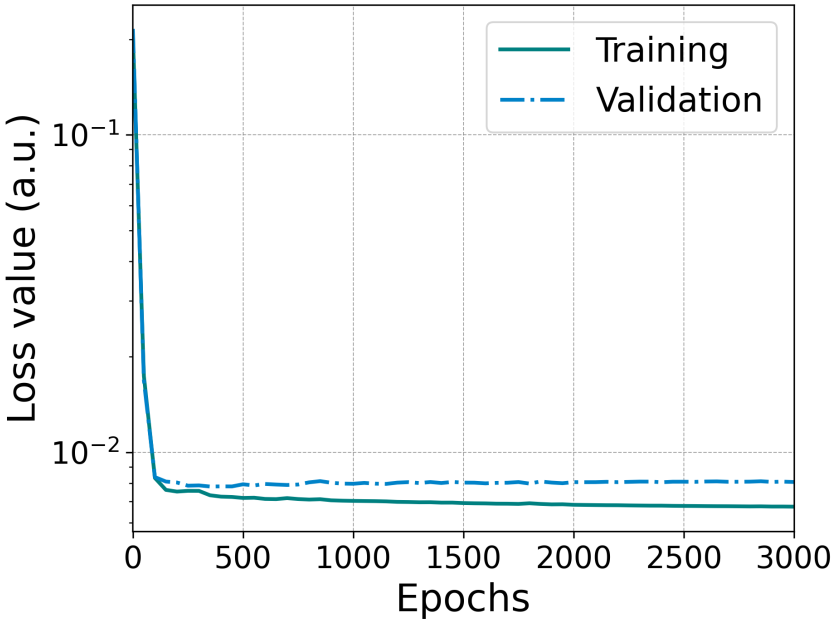

Fig. S4: Training loss (a.u.) of the Local Energy Loss (LES) based machine-learned energy density from regularized second-order Møller–Plesset perturbation theory (ML2) as a function of training steps (epochs). The training dataset is H^-, He, Be, Mg, Ne, Ar, Ca, and Kr. The validation dataset is Li^+, Ca^{2+} and Ne^{2+}.

In Fig. **S5**, we show the absolute global energy error result for GES-based ML2 as a counterpart to Fig. **3b**. Due to the sparse training dataset for the GES, our NN prediction of correlation energies gets worse at larger interatomic distances with increasing training epochs. Furthermore, the energy error is one order of magnitude higher in comparison to the results from the LES-based predictions in Fig. **3b**, underscoring again the advantage of LES over GES.

To study the learning process of the ML2 model, we define the following Relative Loss:

$$\text{Relative Loss} \sim \left| \frac{\text{Loss(epochs)}}{\text{Loss(zeroth epoch)}} \right| \times 100, \tag{S22}$$

In Fig. **S6**, we plot the Relative Loss with respect to training steps (epochs) of the GES-based and LES-based ML2 training. LES shows much faster and smoother convergence compared to the GES counterpart. This is another crucial advantage of LES over GES, in addition to data efficiency.

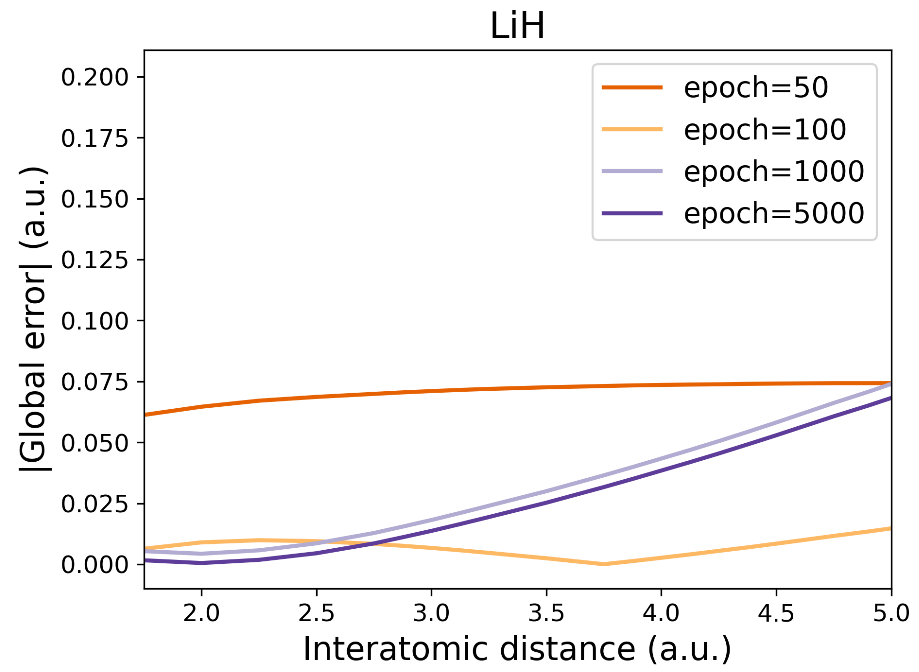

Fig. S5: Absolute Global error in a.u. of the Global Energy Loss (GES) based machine-learned results from regularized second-order Møller–Plesset perturbation theory (ML2) as in Fig. **3b**.

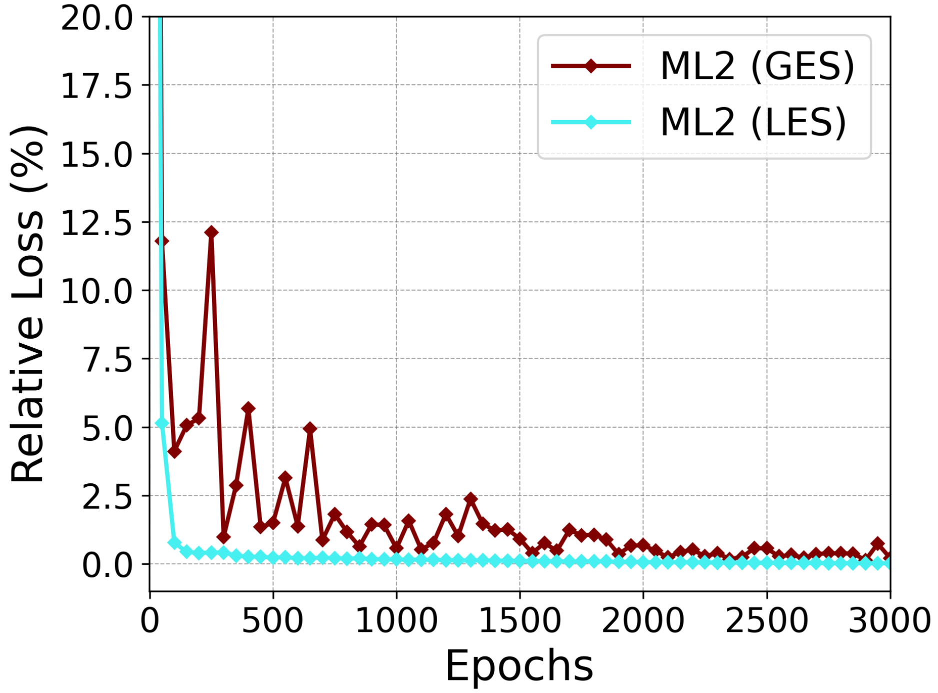

Fig. S6: Relative Loss (see Eq. S22) of Local Energy Loss (LES) based and Global Energy Loss (GES) based machine-learned results from regularized second-order Møller–Plesset perturbation theory (ML2) with respect to the initial loss value.

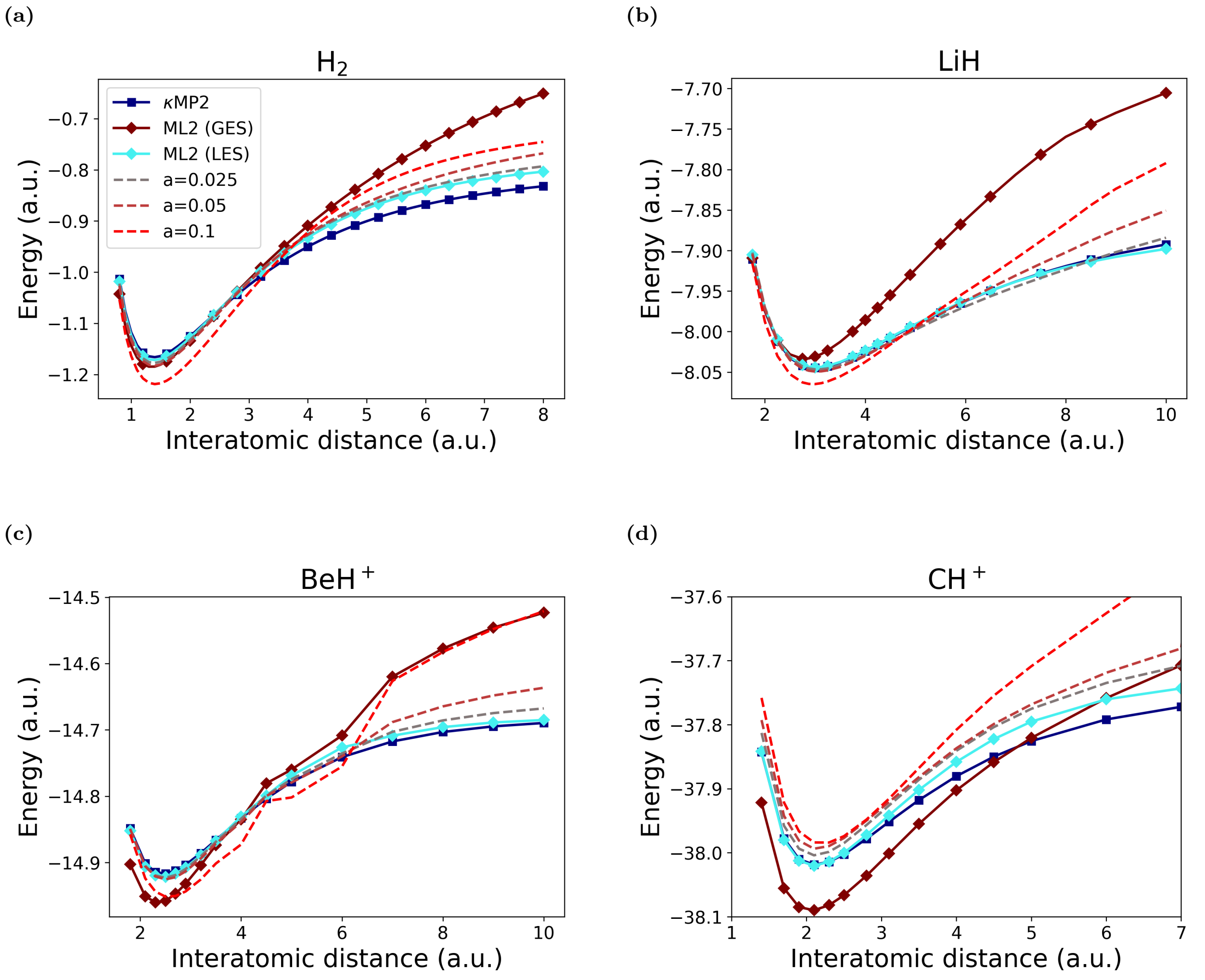

Fig. S7: Dissociation curves as in Fig. 4a for additional diatomic systems. Shown are machine-learned results (ML2) from κ-regularized second-order Møller–Plesset perturbation theory (κMP2) using Global Energy Loss (GES) or Local Energy Loss (LES). Additional LES-based ML2 energies come from neural networks that employ LES with proxy correlation energy densities at different $a-$parameter dependent gauges (see Eq. 15). Since the $w_{\mathrm{c}}(\mathbf{r})$ weights from Eq. 14 for $|a| > 0$ are not necessarily bounded between -1 and 1, we learn them as $x(\mathbf{r}) = \tanh\left(\frac{e_{\mathrm{c}}^{a}(\mathbf{r})}{e_{\mathrm{x}}(\mathbf{r})\rho^{-1/3}(\mathbf{r})}\right)$, retrieving machine-learned correlation energies by applying $\tanh^{-1}$ to $(x(\mathbf{r}))$, which numerically reduces to ML2 (LES) when $a = 0$.

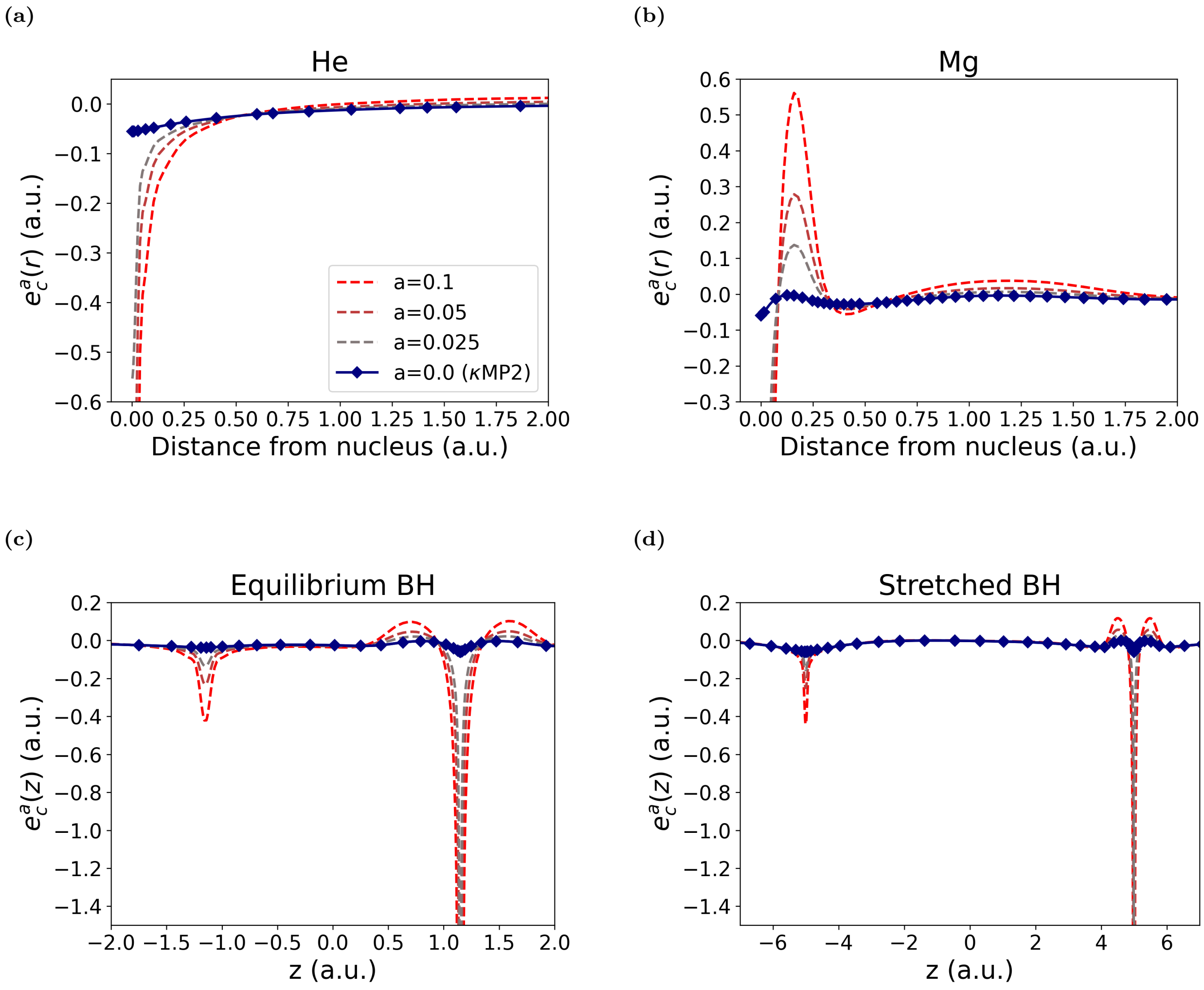

Fig. S8: Gauge transformed correlation energy densites per particle ($e_c^a(r)$ from Eq. (15)). For $a = 0.0$ the result reduces to κ-regularized second-order Møller–Plesset perturbation theory (κMP2). **(a) and (b): Atoms used in Training.** $e_c^a(r)$ as in Fig. **1(c,top)** for the helium atom and the magnesium atom at different a values. **(c) and (d): Diatomics used in Testing.** $e_c^a(z)$ as in Fig. **1(d,top)** along the principle axis at different a values for equilibrium (2.3 a.u. interatomic distance) and stretched (10.0 a.u. interatomic distance) BH.

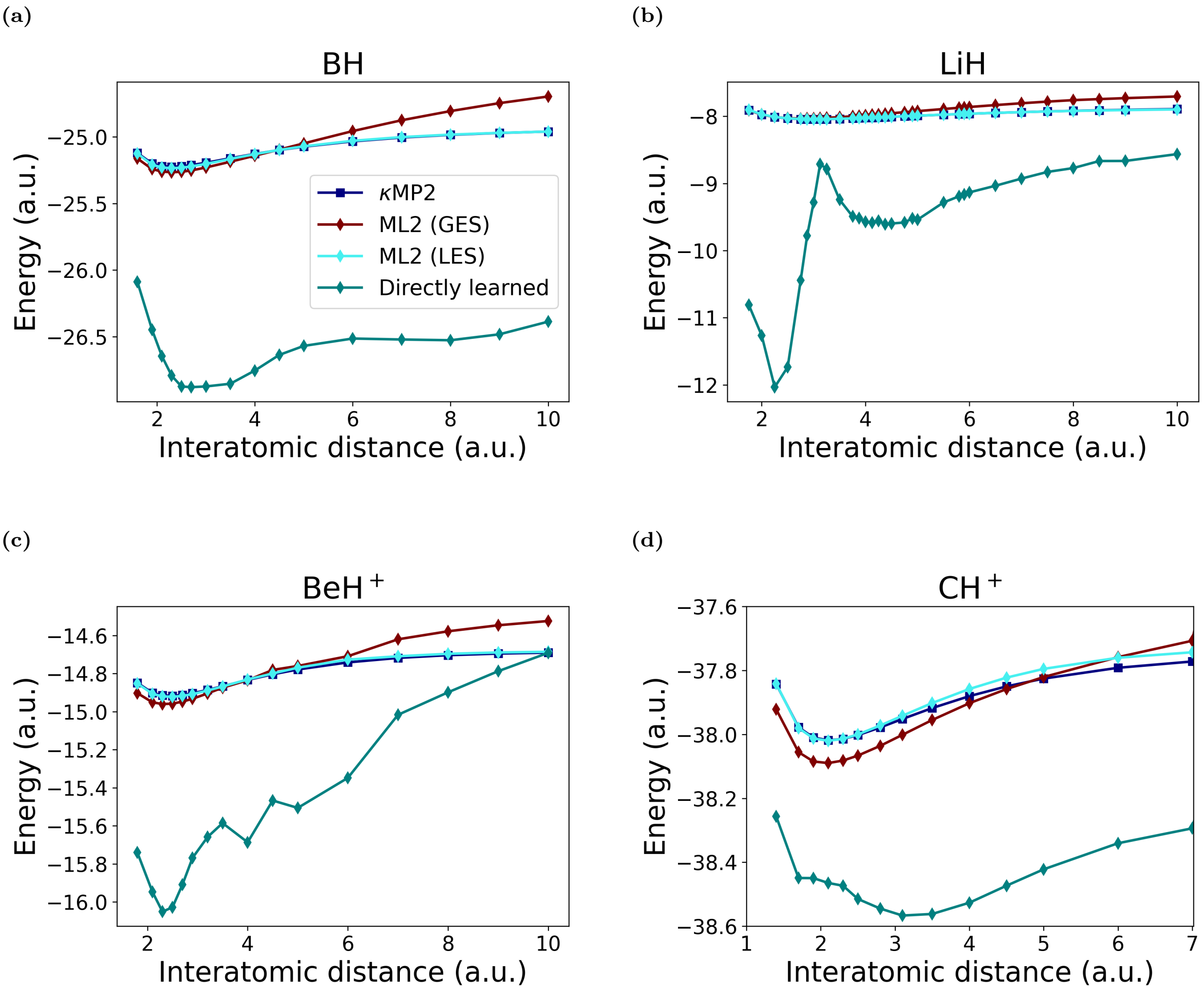

Fig. S9: Dissociation curves as in Fig. 4b for additional diatomic systems. The machine-learned results (ML2) from κ-regularized second-order Møller–Plesset perturbation theory (κMP2) are based on the Global Energy Loss (GES) or Local Energy Loss (LES) learned weights in Eq. 14, while the directly learned predictions adopt a 'direct loss' (see the main text) and learn the neural network weights via tanh preprocessing as in Fig. **S7**.

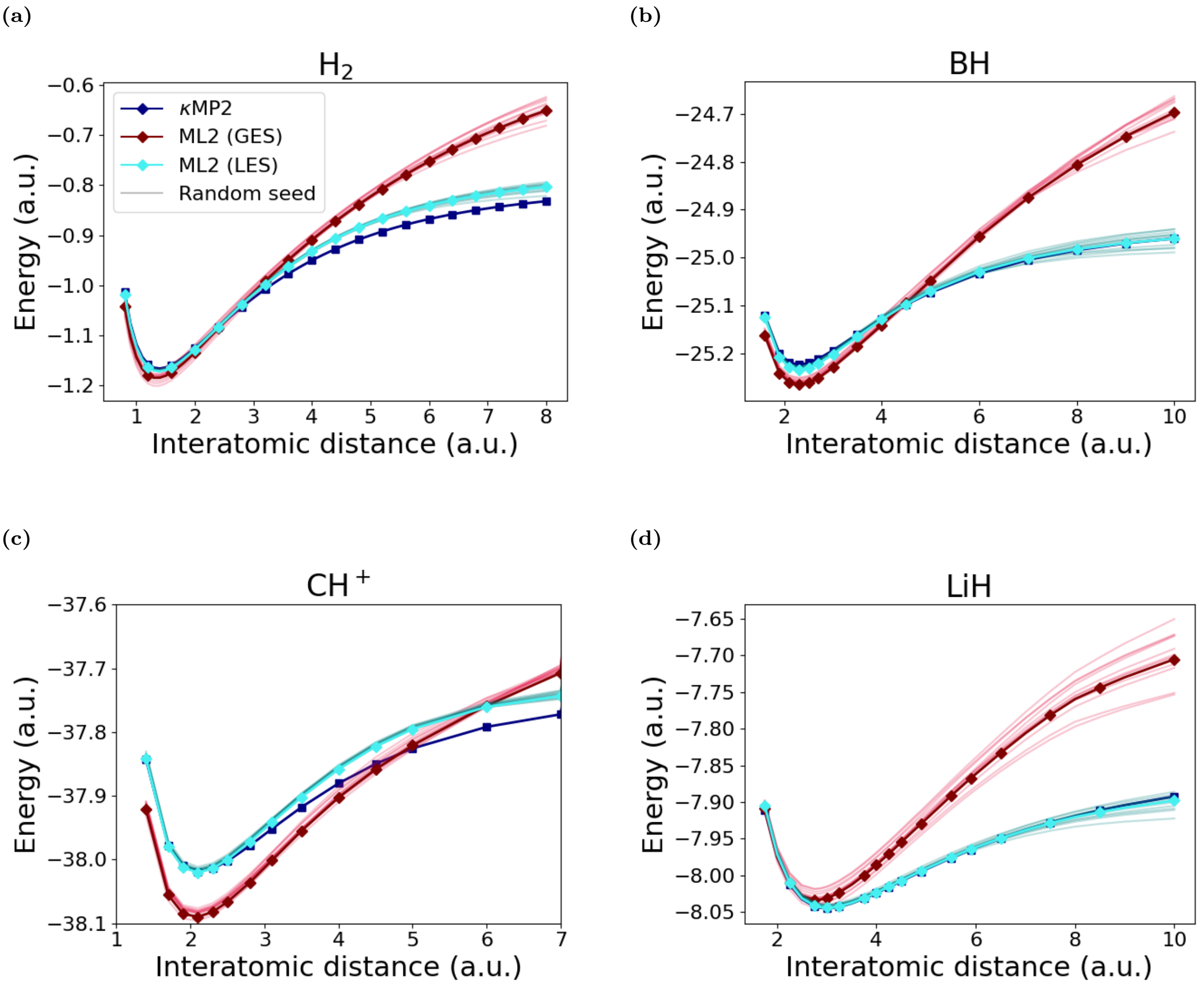

Fig. S10: Dissociation curves as in Fig. 4c for additional diatomic systems. Shown are machine-learned results (ML2) from κ-regularized second-order Møller–Plesset perturbation theory (κMP2) using Global Energy Loss (GES) or Local Energy Loss (LES). Random seed results correspond to GES-based and LES-based ML2 predictions coming from different random seed initializations.

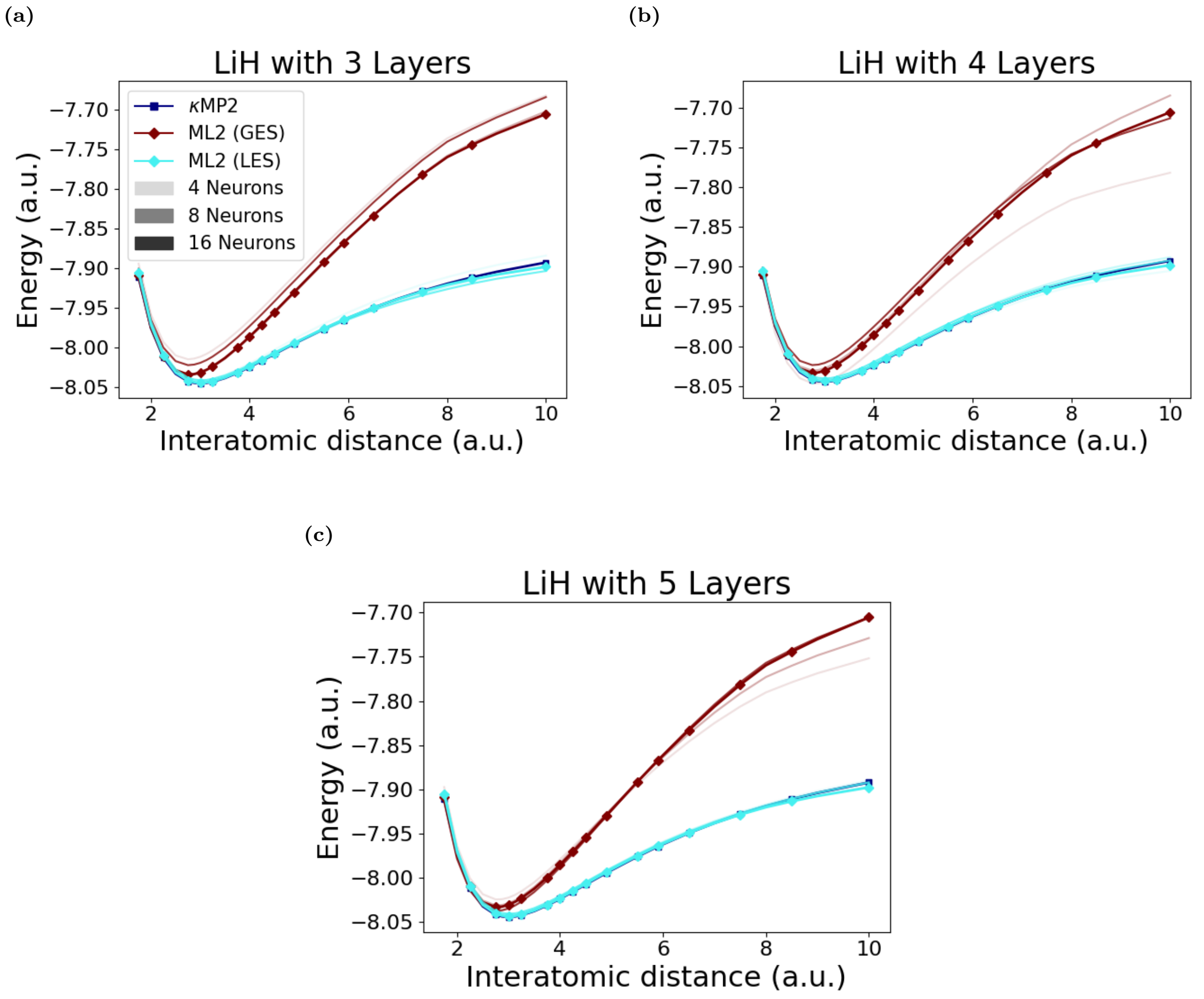

Fig. S11: Dissociation curves as in Fig. 4d with different neural network architectures. Shown are machine-learned results (ML2) from κ-regularized second-order Møller–Plesset perturbation theory (κMP2) using Global Energy Loss (GES) or Local Energy Loss (LES). Additional GES-based and LES-based ML2 predictions come from different neural network architectures, where the number of neurons are given per layer.

In Fig. **S12**, we show dissociation curves for six diatomic systems similar to Fig. **1(c,bottom)**. Here, the GES-based and LES-based ML2 predictions are supplemented by ML2 results coming from a squared LES (see Eq. 3),

$$\mathcal{L}_{\mathrm{LES}}^{2} \sim \int |e^{\mathrm{ref}}(\mathbf{r}) - e^{\mathrm{ML}}(\mathbf{r})|^{2} \rho(\mathbf{r})\, d\mathbf{r}. \tag{S23}$$

We can observe in all plots in Fig. **S12** that the transferability of the LES-based ML2 is not affected by the choice of the loss function.

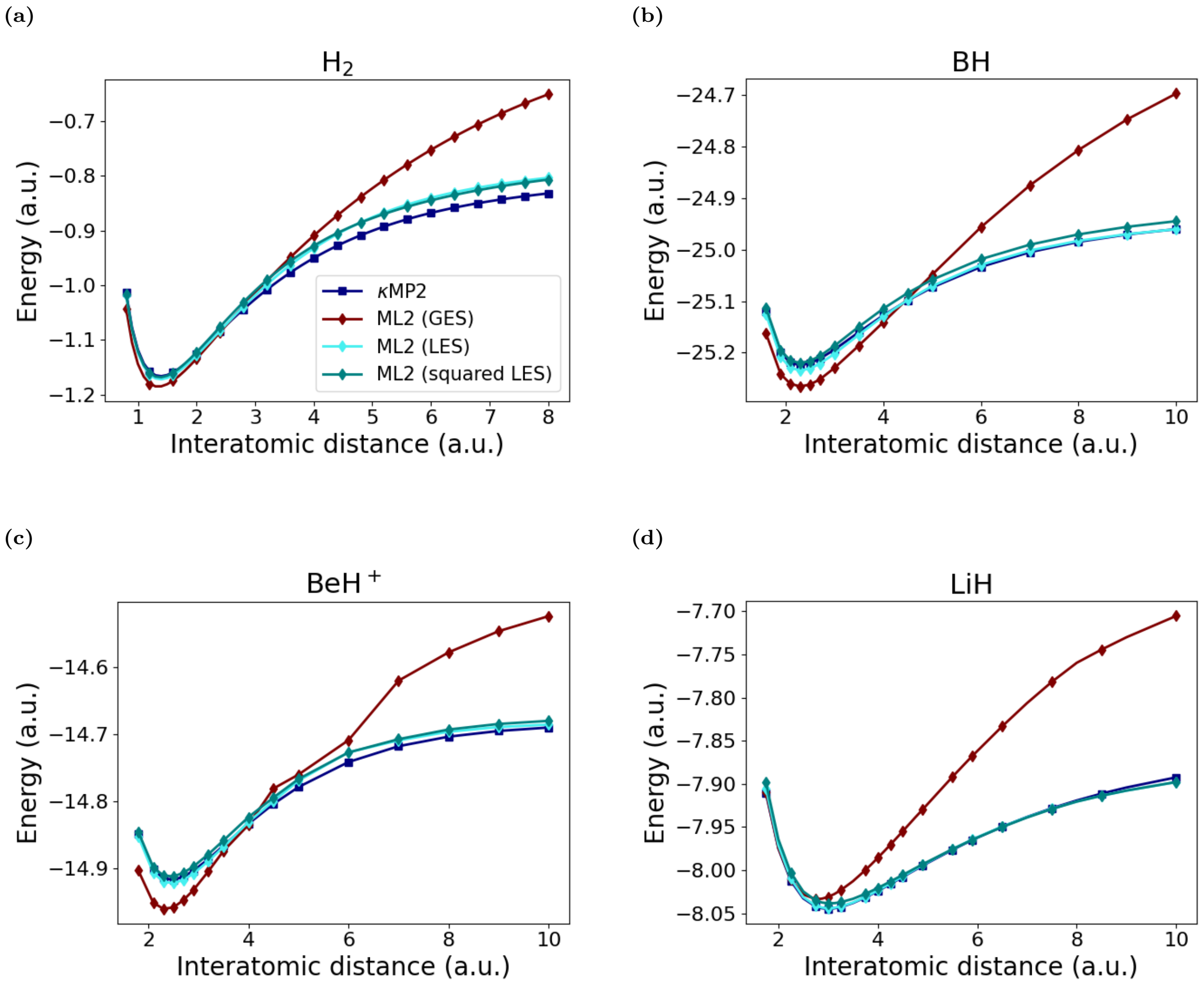

Fig. S12: Dissociation curves as in Fig. 1(c,bottom) for additional diatomic systems. Shown are machine-learned results (ML2) from κ-regularized second-order Møller–Plesset perturbation theory (κMP2) using Global Energy Loss (GES) or Local Energy Loss (LES). ML2 energy predictions in teal diamonds come from a squared LES training strategy (see Eq. S23).

S10. TRAINING SET SIZE DEPENDENCE ON ML2 TRANSFERABILITY TO DIATOMICS

The dissociation curve of BeH^+ in Fig. **S13a** compares the proxy reference, κMP2 and the GES-based and LES-based ML2 models once more data is progressively added to the training. Each "ML2 (GES)+" curve corresponds to a training set containing of the included eight small closed-shell atoms/ions and seven extra energy data points coming from geometries at and around the equilibrium structure of the labeled diatomic systems. These are (in order) H_2, LiH, Li_2, BH, CH^+, N_2 and BeH^+. This means that at each step (each new addition), the NNs are retrained and applied to dissociation curves. While the final training set and additions at each step may not be large in absolute terms, they are major relative to the test (e.g., for the full BeH^+ dissociation curve test, the final model includes in the training its own BeH^+ points near-equilibrium) and the original atomic training set.

LES-based ML2 trained on atoms already gives highly accurate dissociation curves and shows virtually no changes as more training data is added to LES-based ML2. In contrast, GES-based ML2, even after these extra points are added to the training set, is still outperformed by LES. While GES-based curves are improving after more data are added to the training, one can still observe unphysical discontinuities and "bumps"[18] in some of the GES-based curves. In the final batch, we construct the most data-rich model, which even includes equilibrium BeH^+ datapoints in its own training set. Yet, despite this expanded training, the GES-based ML2 remains insufficiently accurate for stretched BeH^+ geometries, unlike the LES-based approach. Using these same models with increasing training data and applying them to the dissociation curves of the other three dimers shown in Fig. **S13a**, similar trends are observed.

Finally, it should be noted that although the GES-based ML2 employs the global energy loss (Eq. 2) for training, it still uses the ML2 energy density per particle ansatz given by Eq. 14. This ansatz has been constructed based on the scaling constraint (Eq. 13) satisfied by $e_c^{\kappa\text{MP2}}(\mathbf{r})$ within our chosen gauge. Consequently, GES-based ML2 is still partially informed by our energy densities per particle gauge, which in turn improves its transferability. Crucially, if instead of this GES that we use here, one employs "raw" GES—i.e., using Eq. 2 to learn energy densities without the ansatz of Eq. 14, the resulting GES transferability would be even worse than shown here.

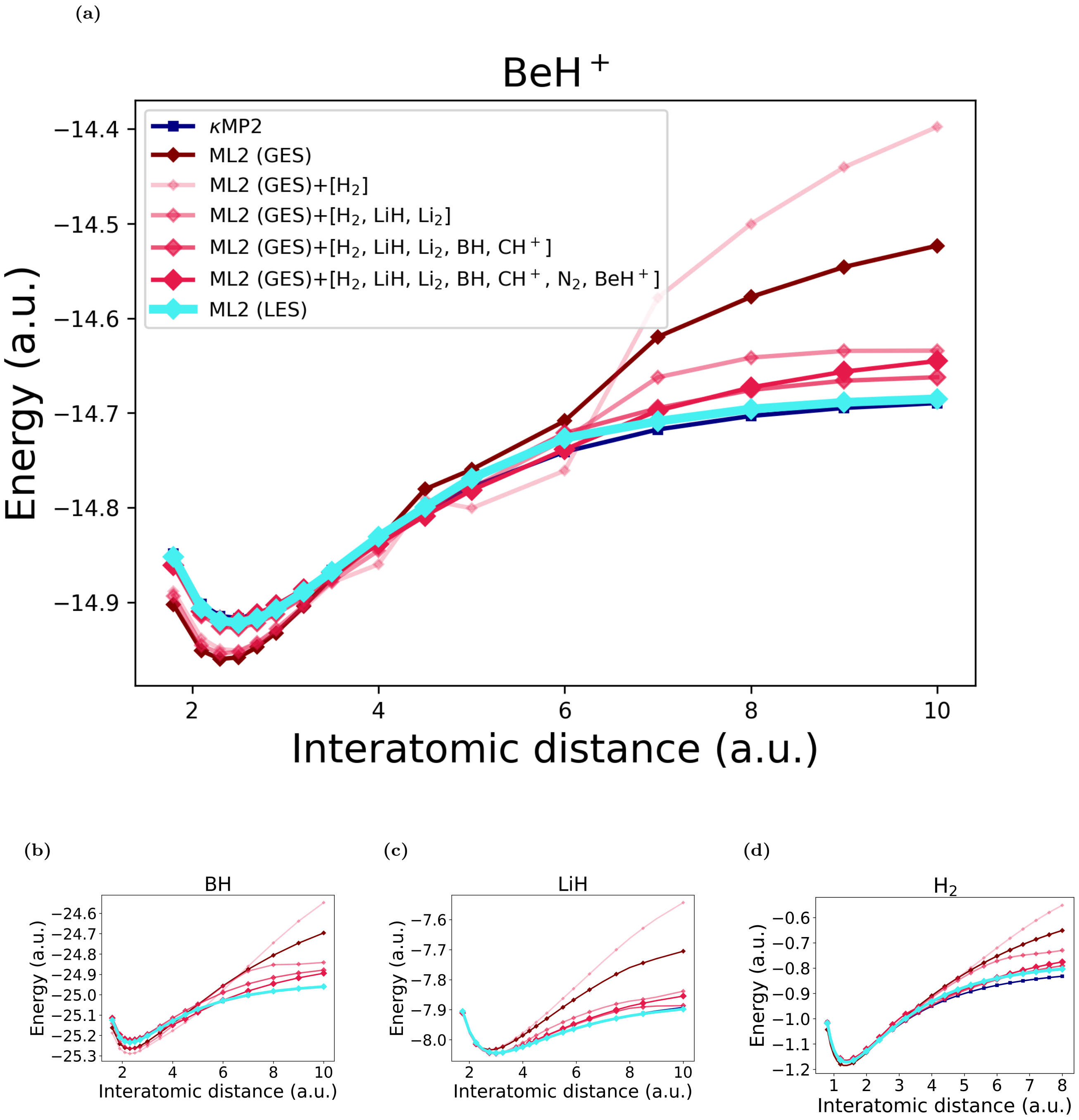

Fig. S13: Dissociation curve of BeH$^+$ as in Fig. 1(c,bottom). Shown are machine-learned results (ML2) from κ-regularized second-order Møller–Plesset perturbation theory (κMP2) using Global Energy Loss (GES) or Local Energy Loss (LES). Additional GES-based ML2 energy predictions come from neural networks obtained by progressively increasing training set size. For each labeled diatomic molecule, seven energy training datapoints near its equilibrium geometry are added to the training set.

S11. SCALING OF MLS2 WEIGHTS

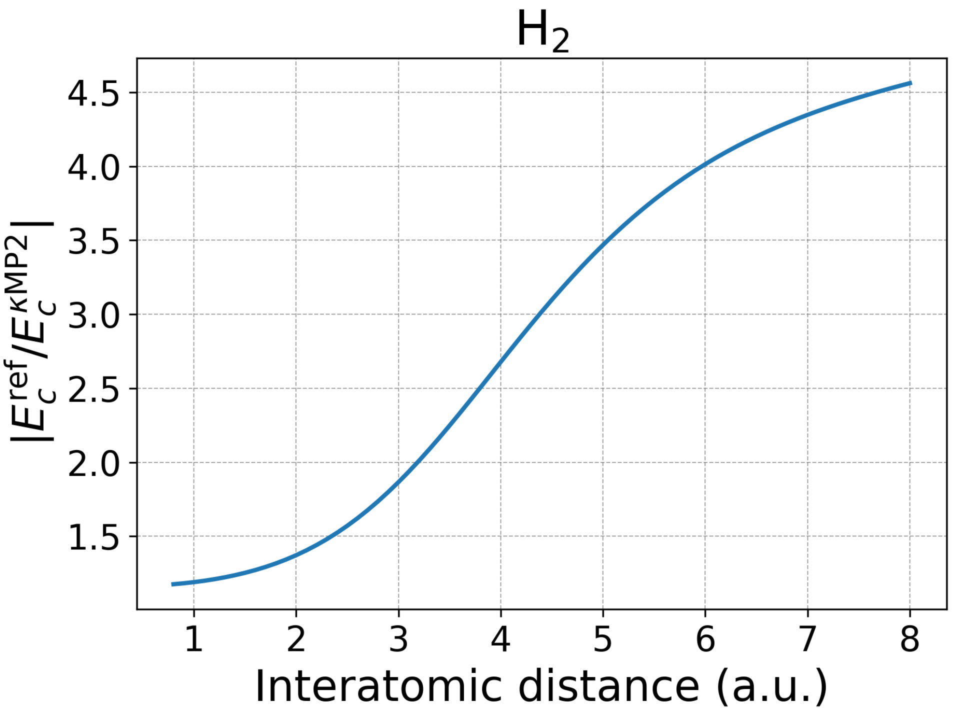

Fig. S14: Absolute ratio between correlation energies. Plotted is the ratio of full configuration interaction (ref) energies and κ-regularized second-order Møller–Plesset perturbation theory (κMP2) energies for H_2 as a function of interatomic distance.

The scaling of the machine-learned weights in Eq. 16 is important for the ability to predict correlation energies when the true value is much greater than those of κMP2. This situation is particularly pronounced when stretching bonds, see Fig. **S14**, where the ratio between the true and κMP2 correlation energies easily exceeds a factor of 4 once the H_2 bond is stretched. Without this adjustment, the MLS2's correlation energies could never be larger than those of κMP2, as $\mathrm{w}_{\mathrm{os}}(\mathbf{r})$ and $\mathrm{w}_{\mathrm{ss}}(\mathbf{r})$ derived from the sigmoid activation function are bound between 0 and 1.

S12. TRAINING AND TEST DATASETS OF MLS2

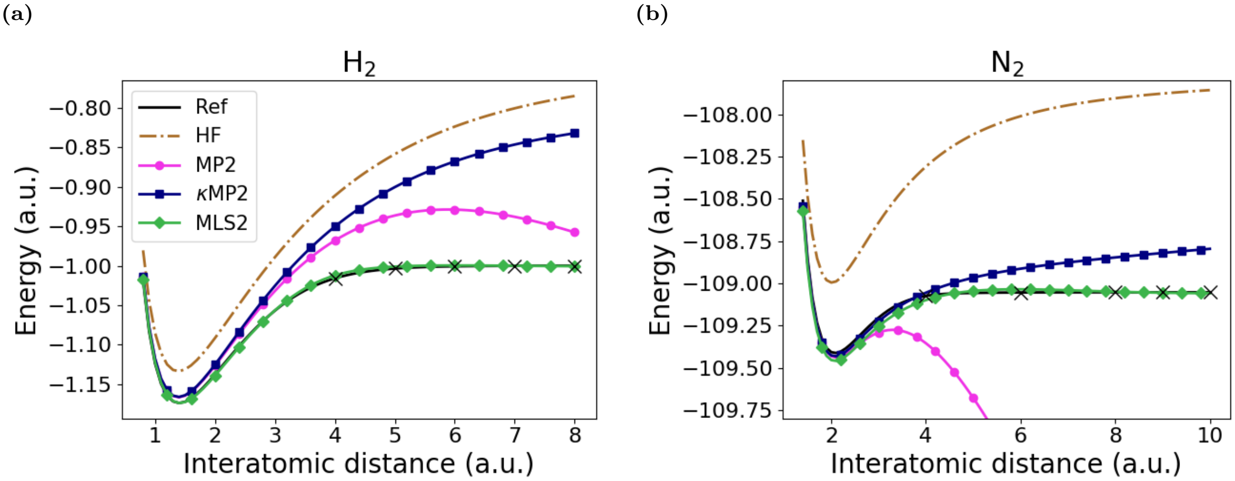

Fig. S15: Dissociation energy curves as in Fig. 5. Hartree-Fock (HF), second-order Møller–Plesset perturbation theory (MP2) and its κ-regularized counterpart (κMP2) are compared with results from the real-space, machine-learned, and regularized extension of spin-component-scaled second-order Møller–Plesset perturbation theory (MLS2). MLS2's energy training data is indicated as the cross-marked points on the black curve. **(a):** Dissociation energy curve of H_2 with reference (Ref) values from full configuration interaction. **(b):** Dissociation energy curve of N_2 with Ref values taken from Ref. 5.

For correlation energy data, the same eight small closed-shell atoms/ions (H^-, He, Be, Mg, Ne, Ar, Ca, and Kr) as for ML2 are employed. Additionally, the following collection of 13 small closed-shell complexes from the MB16-43 database[19] is incorporated: Cl_2, BeH_2, NaH, H_2, BH_3, AlH_3, MgH_2, N_2, SiH_4, LiH, F_2, CH_4 and P_2. Here, we calculate the reference correlation energies with CCSD(T)/*def2-QZVP* in PySCF[6,7] for structures taken from the original dataset. We further train the MLS2 model on correlation energy data points from H_2 (calculated at full configuration interaction level of theory), N_2 (taken from Ref. 5) and Li_2 (taken from Ref. 20) at five different large interatomic distances, namely (in a.u.) $4, 5, 6, 7$ and 8 for H_2 (equilibrium at 1.4), $4, 6, 8, 9$ and 10 for N_2 (equilibrium at 2.1) and $5, 6, 7, 8.5$ and 10 for Li_2 (equilibrium at 5.05). They are visualized, for instance, as cross-marked points in Fig. **S15**. We also plot the HF, MP2, and κMP2-based results for comparison in Figs. **S15a** and **S15b** respectively. Adding the training data points at large distances helps MLS2 to efficiently bridge the gap between regularized PT2 and true correlation energies.

Finally, the reference data for the interaction energy training is obtained from all 18 dispersion-bonded complexes in the RG18 dataset[21]. We employ the interaction correlation energies for the loss in Eq. S21 coming from the difference of HF interaction energies and the database reference values, which are obtained at the CCSD(T) level of theory using the complete basis set limit[22,23] (CBS).

From the W4-11 database[24], we select for correlation energy testing the following subset of 96 closed-shell systems that are not included in our training set from above: AlF_3, S_4, cis-N_2H_2, HN_3, HONC, trans-N_2H_2, HF, HCL, HCN, ethanol, CF_4, BeF_2, AlH, CS_2, Cl_2O, P_4, HNC, S_2O, CH_2F_2, CH_2NH, BH, CCl_2, N_2H_4, SO_3, F_2O, FCCF, BHF_2, acetic

acid, BF, NCCN, PH_3, Be_2, O_3, B_2H_6, SiH_3F, HCOF, AlF, BN, C_2H_6, CO, Si_2H_6, OCS, H_2O, HOCN, CO_2, allene, SO_2, $BeCl_2$, CF_2, propene, acetaldehyde, NH_3, CS, BF_3, CH_3F, NH_2Cl, trans-HCOH, propane, C_2H_5F, SiF_4, C_2H_3F, H_2CO, oxirane, cis-HONO, formic acid, methanol, $AlCl_3$, S_3, ClCN, CH_2, ClF, HCCF, H_2S, cis-HCOH, HOF, C_2, SiO, C_2H_4, ketene, CH_2C, HCNO, HNO, propyne, oxirene, dioxirane, C_2H_2, CH_3NH_2, trans-HONO, FOOF, N_2O, F_2CO, HNCO, AlCl, HOOH, HOCl and glyoxal. The corresponding reference correlation energies are also obtained with CCSD(T)/*def2-QZVP* in PySCF[6,7].

The BH test data is taken from Ref. 25 and employed similar to the training reference correlation data points of the stretched diatomic systems. Lastly, the interaction energies of the formic acid dimer, and other systems of Fig. **S16** from the S22x5 database[26], are calculated analogously to RG18 at the CCSD(T)/CBS level of theory.

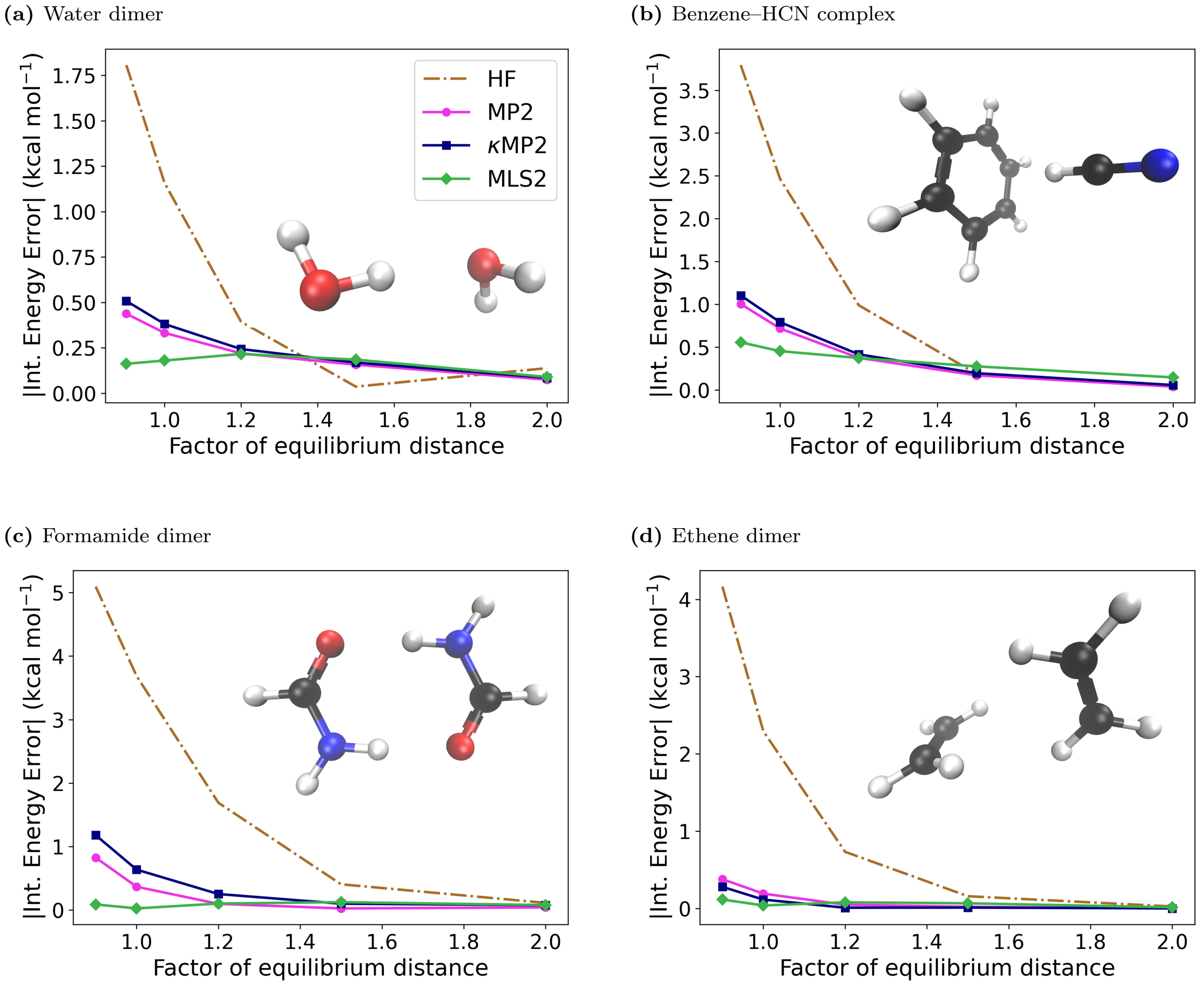

Fig. S16: Interaction (Int.) energy error curves similar to Fig. 1(e,top). Energy errors from Hartree-Fock (HF), second-order Møller–Plesset perturbation theory (MP2) and its κ-regularized counterpart (κMP2) are compared with results from the real-space, machine-learned, and regularized extension of spin-component-scaled second-order Møller–Plesset perturbation theory (MLS2). Reference and geometries taken from the S22x5 database[26].

S13. TRAINING AND TEST DATASETS OF MLS2@W4

For the second MLS2 extrapolation test [MLS2@W4], we employed atomization energies from the W4-11 database[24]. For strictly positive features $r_s(\mathbf{r})$ and $s(\mathbf{r})$ (see Sec. **S6**), we use a $\log(\tanh(\cdot))$ preprocessing. Since the training data consist of atomization energies (interaction), we used the MAE of an interaction-based GES (see Eq. S19) for the training. With 140 training datapoints from W4-11, a randomly chosen subset of 10% (14 atomizations) was used for validation. The MAE calculation for all methods in Fig. **6** considered the following respective reaction energy datapoints: W4-11RE has 11247 total reactions[27], from which 4134 were determined to be SC, SIE4x4 has 16 reactions[28], from which 2 were determined to be SC, BH76 has 76 reactions[29], from which 13 were determined to be SC, BH76RC has 30 reactions[29], from which 1 was determined to be SC and RG18 has 18 reactions[21], from which none was determined to be SC. For the DHs (ωB97M(2)[30] and revDSD-PBEP86-D4[31]), we used total energies from Ref. 32 to compute the MAEs for the individual datasets.

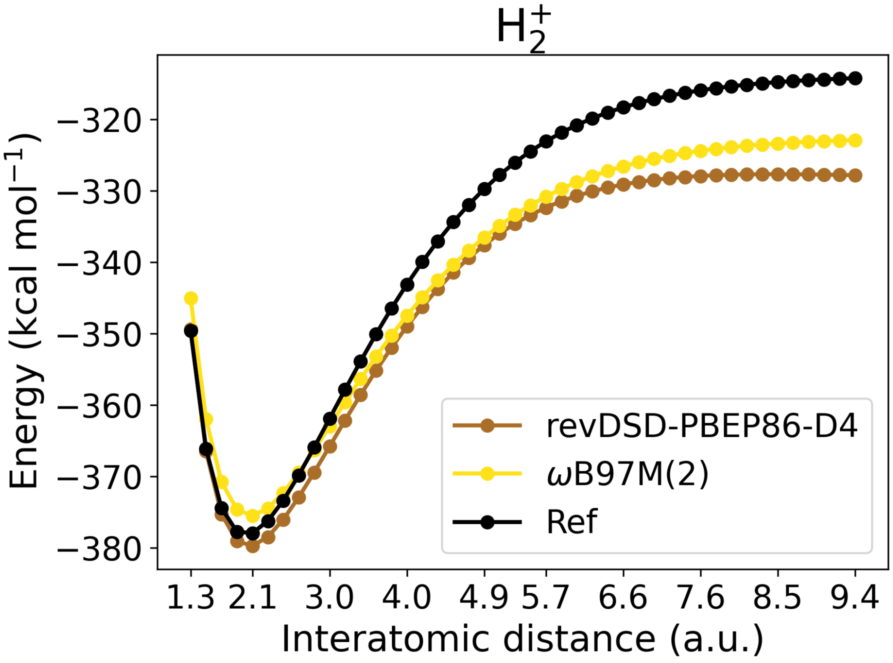

Fig. S17: Dissociation energy curve of H_2^+. Shown are the energy results for ωB97M(2)[30], revDSD-PBEP86-D4[31] and the exact reference (Ref) given by Hartree-Fock (HF), which here is equivalent to second-order Møller–Plesset perturbation theory (MP2), to its κ-regularized counterpart (κMP2) and to the real-space, machine-learned, and regularized extension of spin-component-scaled second-order Møller–Plesset perturbation theory (MLS2): HF = MLS2 = κMP2 = MP2. All evaluated with QChem[33] 6.2.2.

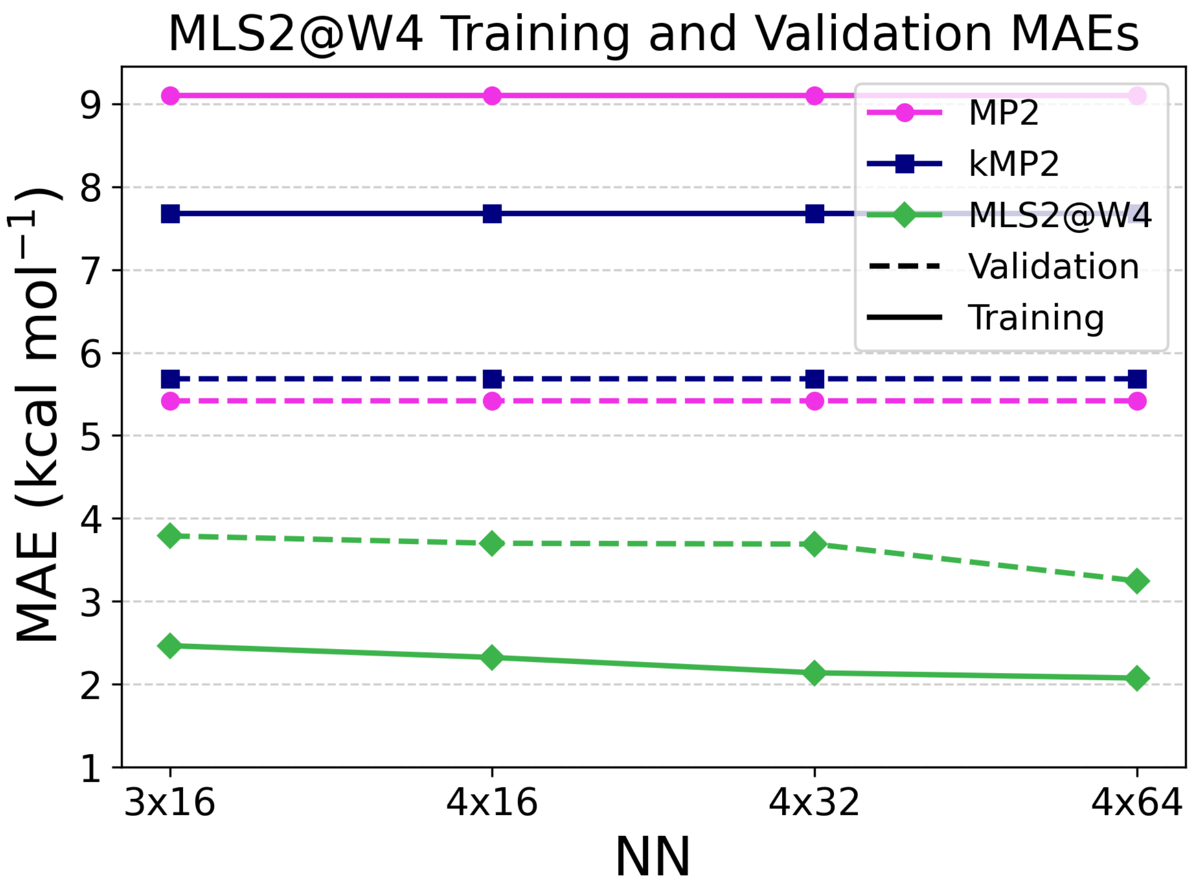

Fig. S18: Energy mean absolute error (MAE) in kcal mol^{-1} as in Fig. 6 for validation (dashed line) and training (solid line) at different neural network (NN) architectures. The real-space, machine-learned, and regularized extension of spin-component-scaled second-order Møller–Plesset perturbation theory (MLS2) model is trained on W4-11 atomization energies[24] (MLS2@W4). MAEs of second-order Møller–Plesset perturbation theory (MP2) and its κ-regularization (κMP2) are shown for comparison. Each NN employs the same training and validation subsets from W4-11, as detailed in Sec. **S13**.

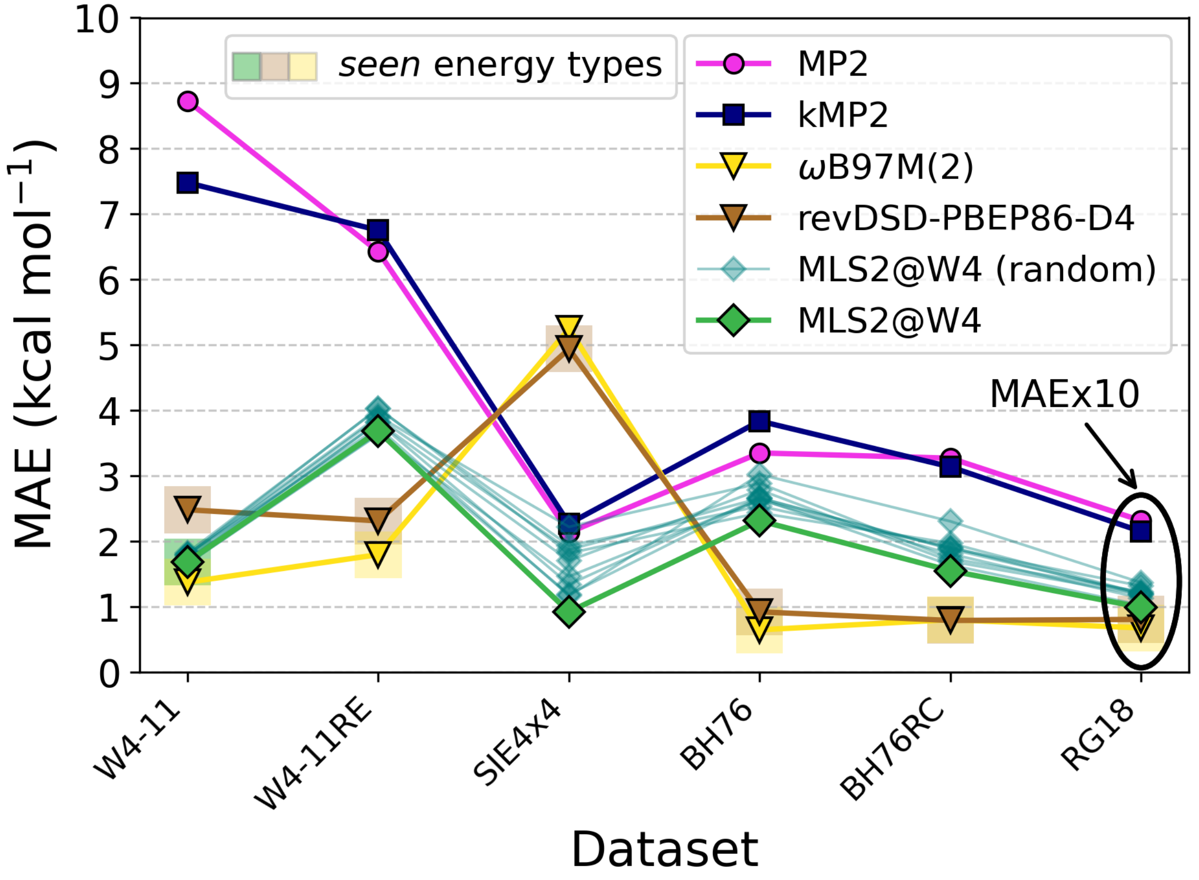

Fig. S19: Energy mean absolute error (MAE) in kcal mol^{-1} as in Fig. 6 with additional results from multiple neural network predictions. The real-space, machine-learned, and regularized extension of spin-component-scaled second-order Møller–Plesset perturbation theory (MLS2) model is trained on W4-11 atomization energies[24] (MLS2@W4). MAEs of second-order Møller–Plesset perturbation theory (MP2), its κ-regularization (κMP2) and of two double-hybrid models (ωB97M(2)[30] and revDSD-PBEP86-D4[31]) are shown for comparison. Translucent squares behind the markers denote seen energy types for the corresponding models (see Methods). The results for RG18 dataset[21] are scaled by a factor of 10 for better visibility. Plotted are 10 additional MAEs of MLS2@W4 predictions coming from neural networks with different seed initializations (random). MLS2@W4 corresponds to the result with the lowest MAE for W4-11.

S14. ADDITIONAL PLOTS FOR ML(S)2 TRAINING DETAILS

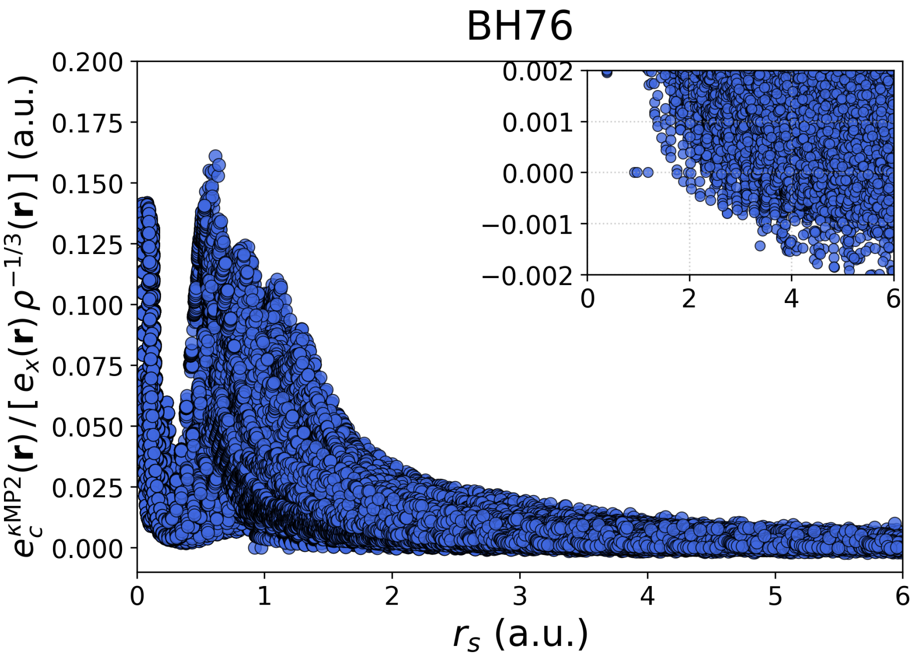

Fig. S20: Scatter plot of "exact" machine-learned weights $w_c(\mathbf{r})$, obtained by inverting Eq. 14, illustrating the range of relevant $w_c(\mathbf{r})$ values from the BH76 dataset[29]. The weights are shown as a function of r_s to highlight energetically important regions ($0 \leq r_s \leq 6$), even though r_s is not used as an input for machine-learning energy densities from regularized second-order Møller–Plesset perturbation theory, for reasons discussed in the main text. The observed range of $w_c(\mathbf{r})$ values, mostly between 0 and 0.2, with some slightly negative values close to zero shown in the inset, motivates the choice of a bounded (tanh) activation function, whose application to the final output layer, with the resulting $(-1, 1)$ output range, readily contains the observed $w_c(\mathbf{r})$ range.

SUPPLEMENTARY REFERENCES

[1]Daas, K.J., Zhao, H., Polak, E. & Vuckovic, S. Exact Møller–Plesset adiabatic connection correlation energy densities. *Journal of Chemical Theory and Computation* **21**, 5501–5513 (2025). doi:10.1021/acs.jctc.5c00348.

[2]Daas, K.J. et.al. Large coupling-strength expansion of the Møller–Plesset adiabatic connection: From paradigmatic cases to variational expressions for the leading terms. *J. Chem. Phys.* **153**, 214112 (2020). doi:10.1063/5.0029084.

[3]Lee, J. & Head-Gordon, M. Regularized orbital-optimized second-order Møller-Plesset perturbation theory: a reliable fifth-order-scaling electron correlation model with orbital energy dependent regularizers. *Journal of Chemical Theory and Computation* **14**, 5203–5219 (2018). doi:10.1021/acs.jctc.8b00731.

[4]Shee, J., Loipersberger, M., Rettig, A., Lee, J. & Head-Gordon, M. Regularized second-order Møller-Plesset theory: a more accurate alternative to conventional mp2 for noncovalent interactions and transition metal thermochemistry for the same computational cost. *The Journal of Physical Chemistry Letters* **12**, 12084–12097 (2021). doi: 10.1021/acs.jpclett.1c03468.

[5]Kats, D. & Tew, D.P. Orbital-optimized distinguishable cluster theory with explicit correlation. *J. Chem. Theor. Comput.* **15**, 13–17 (2019). doi:10.1021/acs.jctc.8b01047.

[6]Sun, Q., Berkelbach, T.C., Blunt, N.S., Booth, G.H., Guo, S., Li, Z., Liu, J., McClain, J.D., Sayfutyarova, E.R., Sharma, S., Wouters, S. & Chan, G.K.-L. PySCF: the Python-based simulations of chemistry framework. *WIREs Compu. Mol. Sci.* **8**, e1340 (2018). doi:10.1002/wcms.1340.

[7]Sun, Q. et.al. Recent developments in the PySCF program package. *J. Chem. Physi.* **153**, 024109 (2020). doi:10.1063/5.0006074.

[8]Dunlap, B.I. Robust and variational fitting. *Phys. Chem. Chem. Phys.* **2**, 2113–2116 (2000). doi:10.1039/b000027m.

[9]Werner, H.J., Manby, F.R. & Knowles, P.J. Fast linear scaling second-order Møller-Plesset perturbation theory (MP2) using local and density fitting approximations. *J. Chem. Phys.* **118**, 8149–8160 (2003). doi:10.1063/1.1564816.

[10]Lebedev, V.I. & Laikov, D.N. A quadrature formula for the sphere of the 131st algebraic order of accuracy. *Doklady Mathematics* **59**, 477–481 (1999). URL `https://api.semanticscholar.org/CorpusID:118893131`.

[11]Hellmann, R., Bich, E. & Vogel, E. Ab initio potential energy curve for the neon atom pair and thermophysical properties of the dilute neon gas. I. Neon-neon interatomic potential and rovibrational spectra. *Mol. Phys.* **106**, 133–140 (2008). doi: 10.1080/00268970701843147.

[12]Jurečka, P., Šponer, J., Černý, J. & Hobza, P. Benchmark database of accurate (MP2 and CCSD(T) complete basis set limit) interaction energies of small model complexes, dna base pairs, and amino acid pairs. *Phys. Chem. Chem. Phys.* **8**, 1985–1993 (2006). doi:10.1039/B600027D.

[13]Furness, J.W. et.al. Accurate and numerically efficient r^2 SCAN meta-generalized gradient approximation. *The Journal of Physical Chemistry Letters* **11**, 8208–8215 (2020). doi: 10.1021/acs.jpclett.0c02405.

[14]Grimme, S. & Hansen, A. A practicable real-space measure and visualization of static electron-correlation effects. *Angew. Chem. Int. Ed.* **54**, 12308–12313 (2015). doi: 10.1002/anie.201501887.

[15]Bauer, C.A., Hansen, A. & Grimme,S. The fractional occupation number weighted density as a versatile analysis tool for molecules with a complicated electronic structure. *Chem. Eur. J.* **23**, 6150–6164 (2017). doi:10.1002/chem.201604682.

[16]Levy, M. & Perdew, J.P. Hellmann-Feynman, virial, and scaling requisites for the exact universal density functionals. Shape of the correlation potential and diamagnetic susceptibility for atoms. *Phys. Rev. A* **32**, 2010–2021 (1985). doi:10.1103/PhysRevA.32.2010.

[17]Van Leeuwen, R. Kohn-Sham potentials in density functional theory. Ph.D. thesis at `https://www.scm.com/wp-content/uploads/thesis.leeuwen.pdf` (1994).

[18]Vuckovic, S., Irons, T.J.P., Savin, A., Teale, A.M. & Gori-Giorgi, P. Exchange-correlation functionals via local interpolation along the adiabatic connection. *Journal of Chemical Theory and Computation* **12**, 2598–2610 (2016). doi:10.1021/acs.jctc.6b00177.

[19]Korth, M. & Grimme, S. "Mindless" DFT benchmarking. *Journal of Chemical Theory and*

Computation **5**, 993–1003 (2009). doi:10.1021/ct800511q.

[20] Lie, G.C. & Clementi, E. Study of the electronic structure of molecules. XXII. Correlation energy corrections as a functional of the Hartree-Fock type density and its application to the homonuclear diatomic molecules of the second row atoms. *J. Chem. Phys.* **60**, 1288–1296 (1974). doi:10.1063/1.1681193.

[21] Grimme, S., Antony, J., Ehrlich, S. & Krieg, H. A consistent and accurate *ab initio* parametrization of density functional dispersion correction (DFT-D) for the 94 elements H-Pu. *J. Chem. Phys.* **132**, 154104 (2010). doi:10.1063/1.3382344.

[22] Halkier, A. et.al. Basis-set convergence in correlated calculations on Ne, N_2, and H_2O. *Chem. Phys. Lett.* **286**, 243–252 (1998). doi:10.1016/S0009-2614(98)00111-0.

[23] Halkier, A. et.al. Basis-set convergence of the energy in molecular Hartree-Fock calculations. *Chem. Phys. Lett.* **302**, 437–446 (1999). doi:10.1016/S0009-2614(99)00179-7.

[24] Karton, A., Daon, S. & Martin, J.M.L. W4-11: a high-confidence benchmark dataset for computational thermochemistry derived from first-principles W4 data. *Chem. Phys. Lett.* **510**, 165–178 (2011). doi:10.1016/j.cplett.2011.05.007.

[25] Mentel, Ł.M., van Meer, R., Gritsenko, O.V. & Baerends, E.J. The density matrix functional approach to electron correlation: dynamic and nondynamic correlation along the full dissociation coordinate. *J. Chem. Phys.* **140**, 214105 (2014). doi:10.1063/1.4879776.

[26] Gráfová, L., Pitoňák, M., Řezáč, J. & Hobza, P. Comparative study of selected wave function and density functional methods for noncovalent interaction energy calculations using the extended S22 data set. *Journal of Chemical Theory and Computation* **6**, 2365–2376 (2010). doi:10.1021/ct1002253.

[27] Margraf, J.T., Ranasinghe, D.S. & Bartlett, R.J. Automatic generation of reaction energy databases from highly accurate atomization energy benchmark sets. *Phys. Chem. Chem. Phys.* **19**, 9798–9805 (2017). doi:10.1039/C7CP00757D.

[28] Goerigk, L. & Grimme, S. A general database for main group thermochemistry, kinetics, and noncovalent interactions – assessment of common and reparameterized (meta-)GGA density functionals. *Journal of Chemical Theory and Computation* **6**, 107–126 (2010). doi:10.1021/ct900489g.

[29] Görigk, L. et.al. A look at the density functional theory zoo with the advanced GMTKN55 database for general main group thermochemistry, kinetics and noncovalent interactions. *Phys. Chem. Chem. Phys.* **19**, 32184–32215 (2017). doi:10.1039/C7CP04913G.

[30] Mardirossian, N. & Head-Gordon, M. Survival of the most transferable at the top of Jacob's ladder: defining and testing the ωB97M(2) double hybrid density functional. *J. Chem. Phys.* **148**, 241736 (2018). doi:10.1063/1.5025226.

[31] Santra, G., Sylvetsky, N. & Martin, J.M.L. Minimally empirical double-hybrid functionals trained against the GMTKN55 database: revDSD-PBEP86-D4, revDOD-PBE-D4, and DOD-SCAN-D4. *J. Phys. Chem. A* **123**, 5129–5143 (2019). doi:10.1021/acs.jpca.9b03157.

[32] Santra, G., Cho, M. & Martin, J.M.L. Exploring avenues beyond revised DSD functionals: I. range separation, with xDSD as a special case. *J. Phys. Chem. A* **125**, 4614–4627 (2021). doi:10.1021/acs.jpca.1c01294.

[33] Shao, Y. et.al. Advances in molecular quantum chemistry contained in the Q-chem 4 program package. *Mol. Phys.* **113**, 184–215 (2015). doi:10.1080/00268976.2014.952696.
